# Supplementary material for: NADPH oxidase 3 inhibition preserves hearing in mice after stereotactic radiosurgery
Source: Mol Ther Nucleic Acids. 2026 Jul 22;37(3):103036. doi: 10.1016/j.omtn.2026.103036 (PMC13487719; doi:10.1016/j.omtn.2026.103036)
Supplement: Supplementary file 1 [file mmc1.pdf]

OMTN, Volume *37*

Supplemental information

NADPH oxidase 3 inhibition preserves hearing in mice after stereotactic radiosurgery

Dimitrios Daskalou, Francis Rousset, Stéphanie Sgroi, Lucie Oberhauser, Jean-Philippe Thiran, Constantin Tuleasca, Ileana O. Jelescu, Marc Levivier, and Pascal Senn

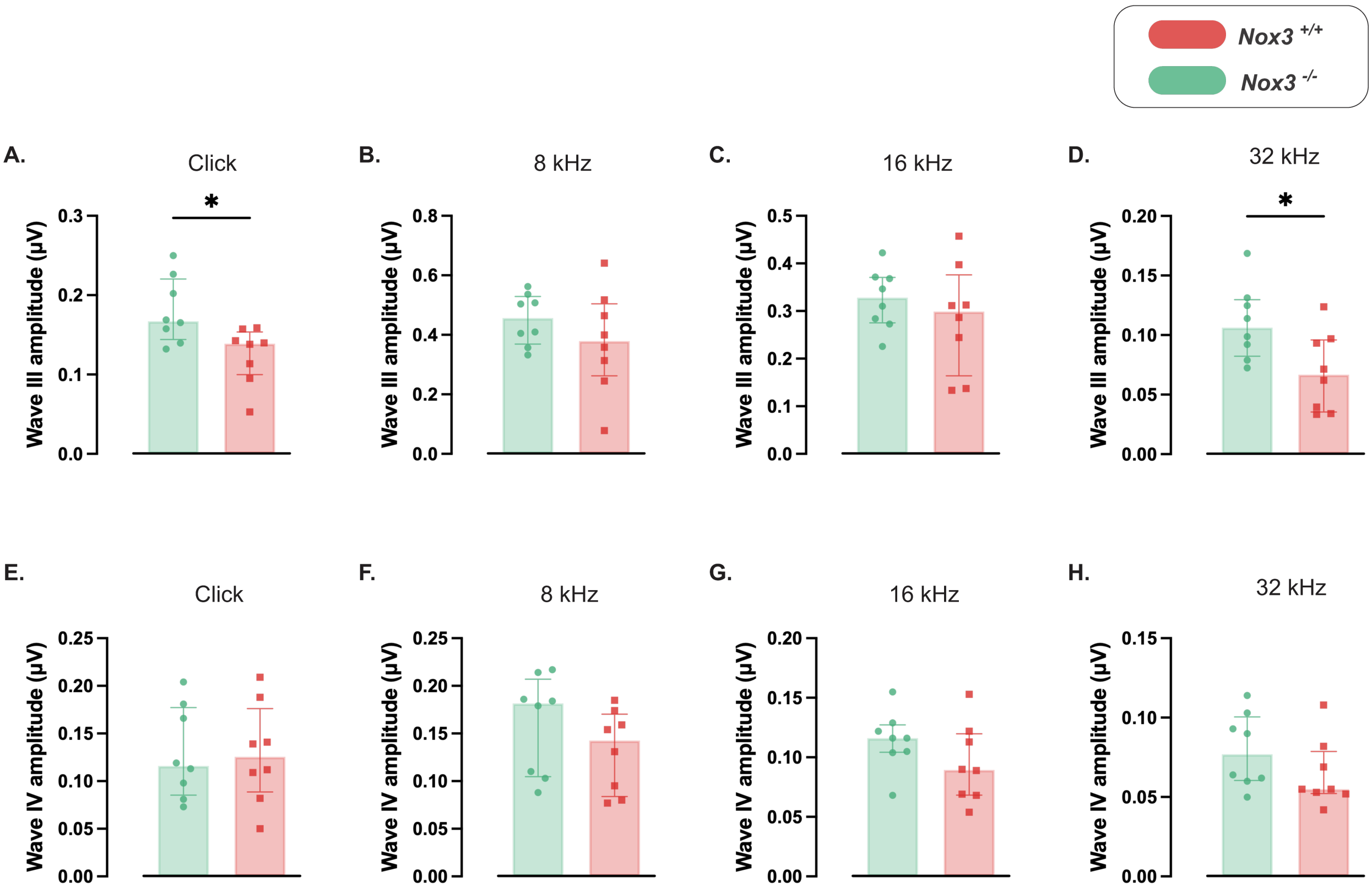

Figure S1 Auditory brainstem response wave III and IV amplitudes at week 4 post-irradiation in wild-type and Nox3-deficient mice.

(A–D) Auditory brainstem response (ABR) wave III amplitudes from the irradiated ears of *Nox3*$^{+/+}$ and Nox3$^{-/-}$ mice (n = 8 per group), measured in response to click (A) and tone bursts at 8 kHz (B), 16 kHz (C), and 32 kHz (D). Median wave III amplitudes were significantly lower in *Nox3*$^{+/+}$ mice after click and 32 kHz stimulation.

(E–H) ABR wave IV amplitudes under the same stimulation conditions. No significant differences were observed between the two groups.

Data are presented as medians with interquartile ranges. Asterisks indicate statistical significance (*$p < 0.05$). Statistical comparisons were performed using Mann–Whitney tests.

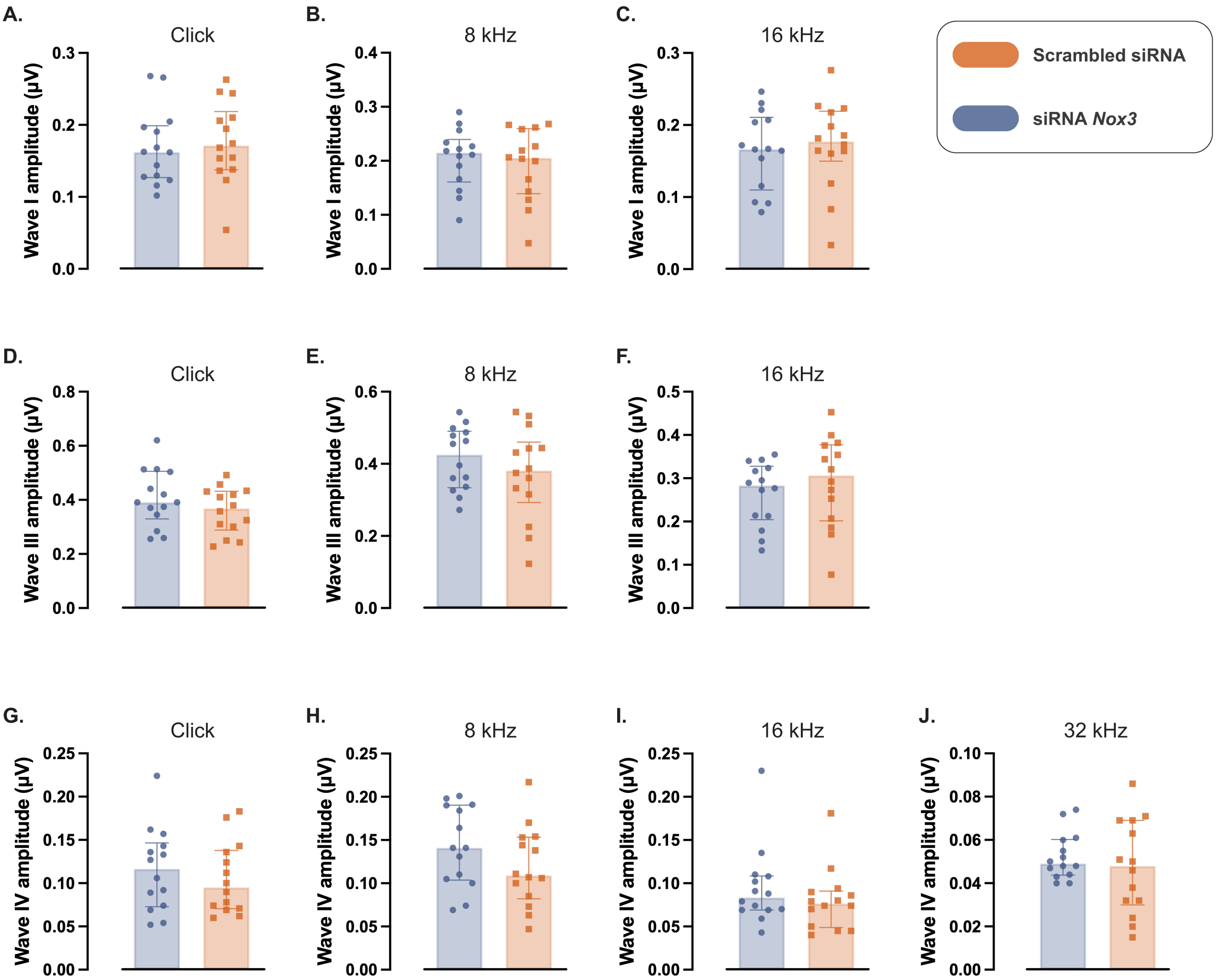

Figure S2 Auditory brainstem response wave I, III, and IV amplitudes at week 4 post-irradiation in siRNA-treated mice.

(A–C) Auditory brainstem response (ABR) wave I amplitudes from the irradiated ears of siScr- and siNox3-treated mice (n = 14 per group), measured in response to click (A) and tone bursts at 8 kHz (B) and 16 kHz (C).

(D–F) ABR wave III amplitudes under the same stimulation conditions.

(G–J) ABR wave IV amplitudes in response to click (G) and tone bursts at 8 kHz (H), 16 kHz (I), and 32 kHz (J).

No significant differences in wave amplitudes were observed between siScr- and siNox3-treated groups except for wave I and III at 32 kHz, which are shown in the main figure (Figures 2F and 2G).

Data are presented as medians with interquartile ranges. Statistical comparisons were performed using Mann–Whitney tests.

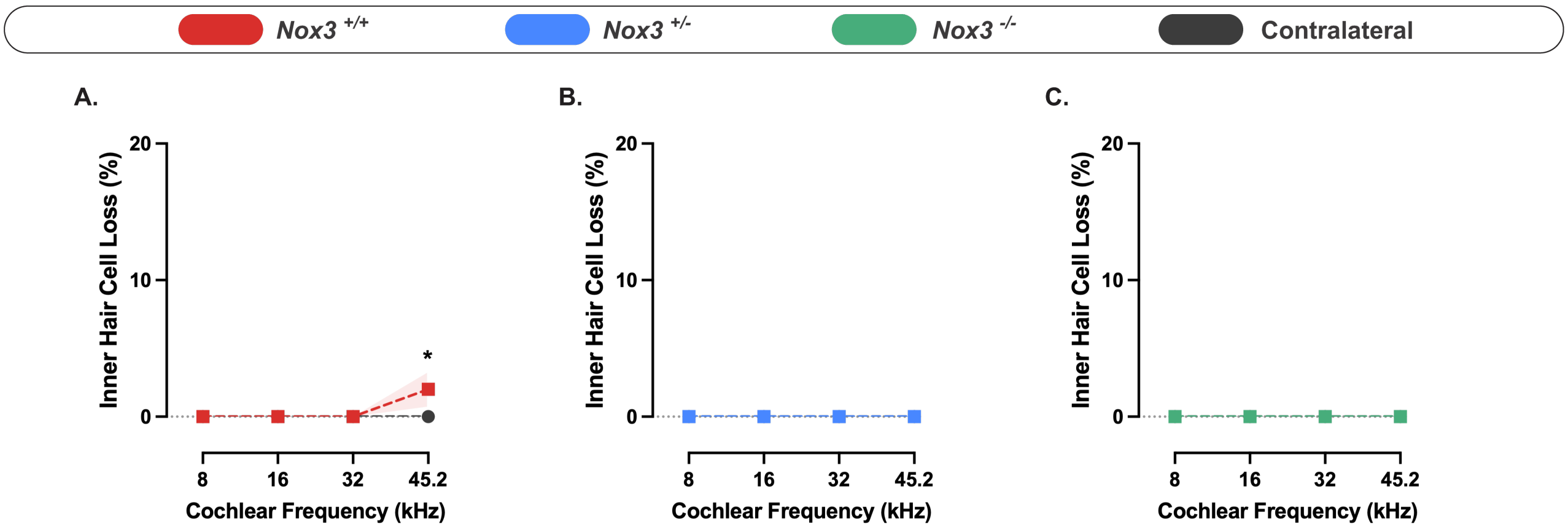

Figure S3 Inner hair cell loss across cochlear frequencies at 4 weeks post-irradiation in Nox3 genotype groups.

(A–C) Percentage inner hair cell (IHC) loss in wild-type (*Nox3*$^{+/+}$; A), heterozygous (*Nox3*$^{+/-}$; B), and *Nox3*-deficient (*Nox3*$^{-/-}$; C) mice (n = 4 per group), relative to the contralateral cochlea. IHCs were preserved in all groups except for a minor loss at 45.2 kHz in *Nox3*$^{+/+}$ mice.

Data are presented as means with shaded areas representing SEM. Asterisks indicate statistical significance (*$p < 0.05$). Statistical comparisons were performed using two-way repeated measures ANOVA with Bonferroni correction for post hoc pairwise comparisons.

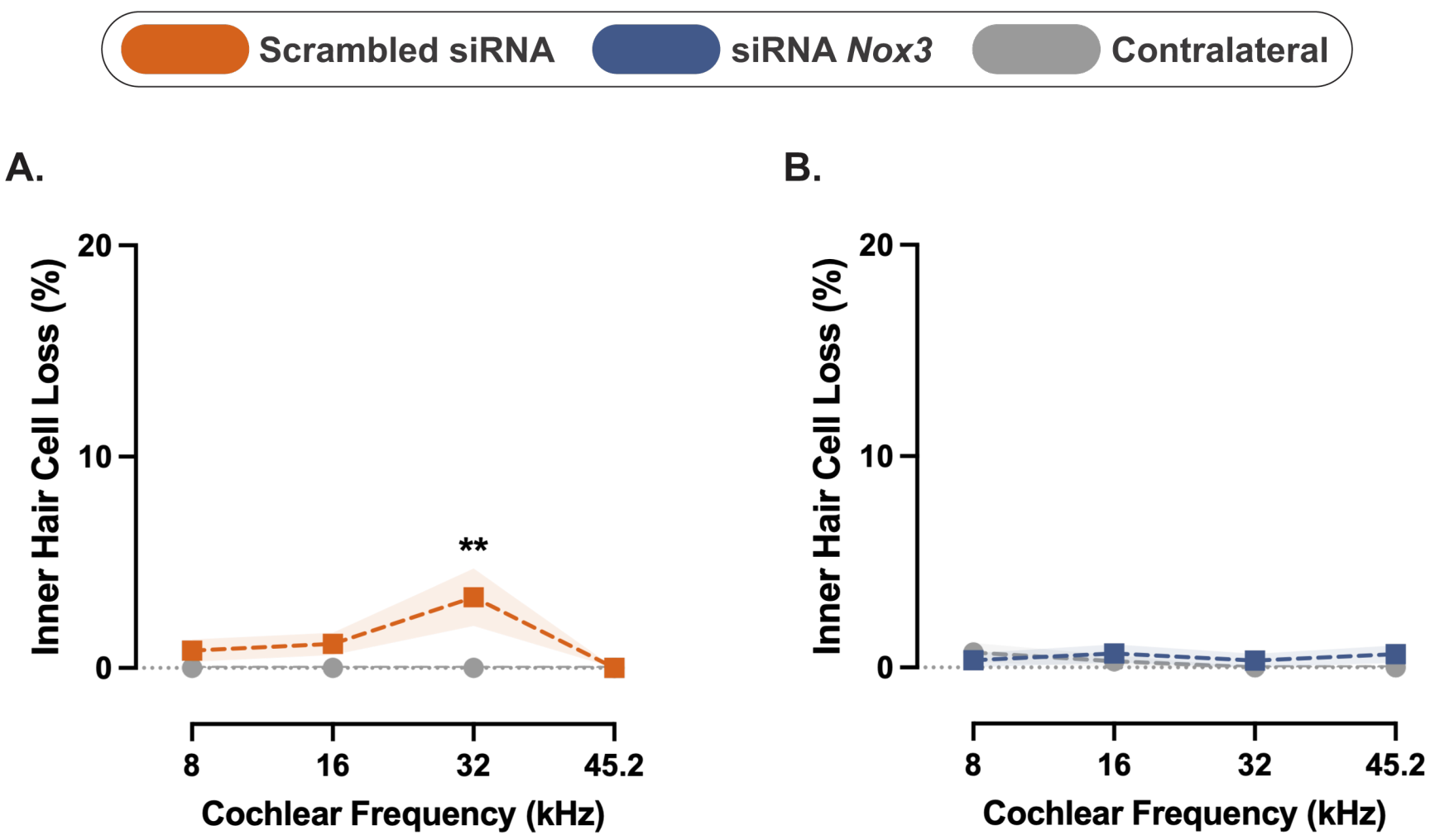

Figure S4 Inner hair cell loss across cochlear frequencies at 4 weeks post-irradiation in siRNA-treated mice.

(A–B) Percentage inner hair cell (IHC) loss in scrambled siRNA (siScr; A; n = 7) and siRNA targeting *Nox3* (siNox3; B; n = 8) groups, relative to the contralateral cochlea. IHCs were preserved in both groups, except for a minor loss at 32 kHz in siScr-treated mice.

Data are presented as means with shaded areas representing SEM. Asterisks indicate statistical significance (**$p < 0.01$). Statistical comparisons were performed using two-way repeated measures ANOVA with Bonferroni correction for post hoc pairwise comparisons.

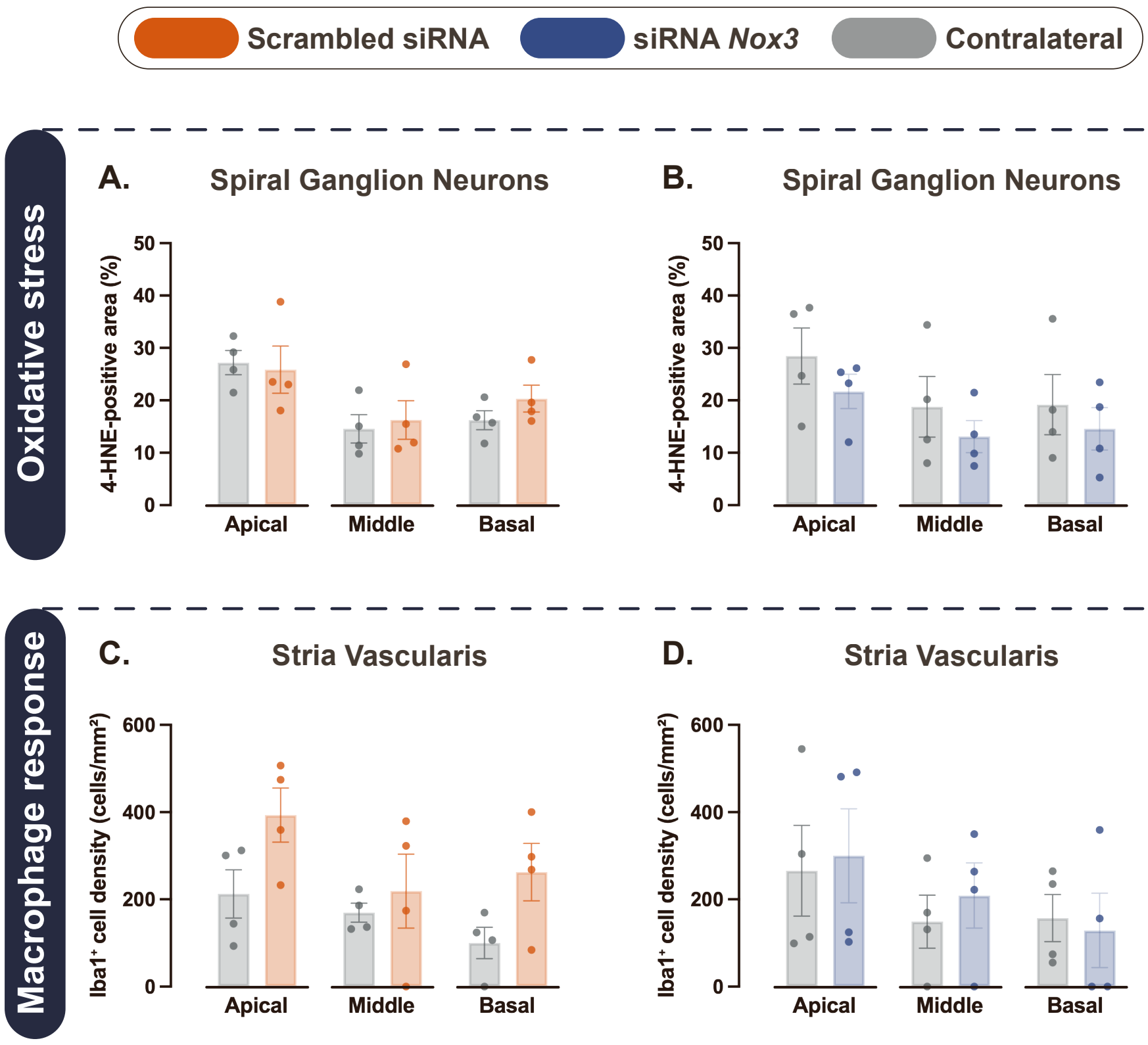

Figure S5 Oxidative stress and macrophage response in spiral ganglion neurons and stria vascularis at 4 weeks post-irradiation in siRNA-treated mice.

(A, B) Percentage of 4-hydroxynonenal (4-HNE)-positive area in spiral ganglion neurons in scrambled siRNA (siScr; A; n = 4) and siRNA targeting Nox3 (siNox3; B; n = 4) groups, relative to the contralateral cochlea. No significant increase in 4-HNE immunoreactivity was observed in either group.

(C, D) Iba1-positive cell density (cells/mm^2) in the stria vascularis in siScr-treated (C; n = 4) and siNox3-treated (D; n = 4) groups, relative to the contralateral cochlea. Macrophage infiltration tended to be higher in siScr-treated mice, although this did not reach statistical significance, whereas no significant increase was detected in siNox3-treated animals.

Data are presented as means ± SEM. Statistical comparisons were performed using two-way repeated measures ANOVA with Bonferroni correction for post hoc pairwise comparisons.

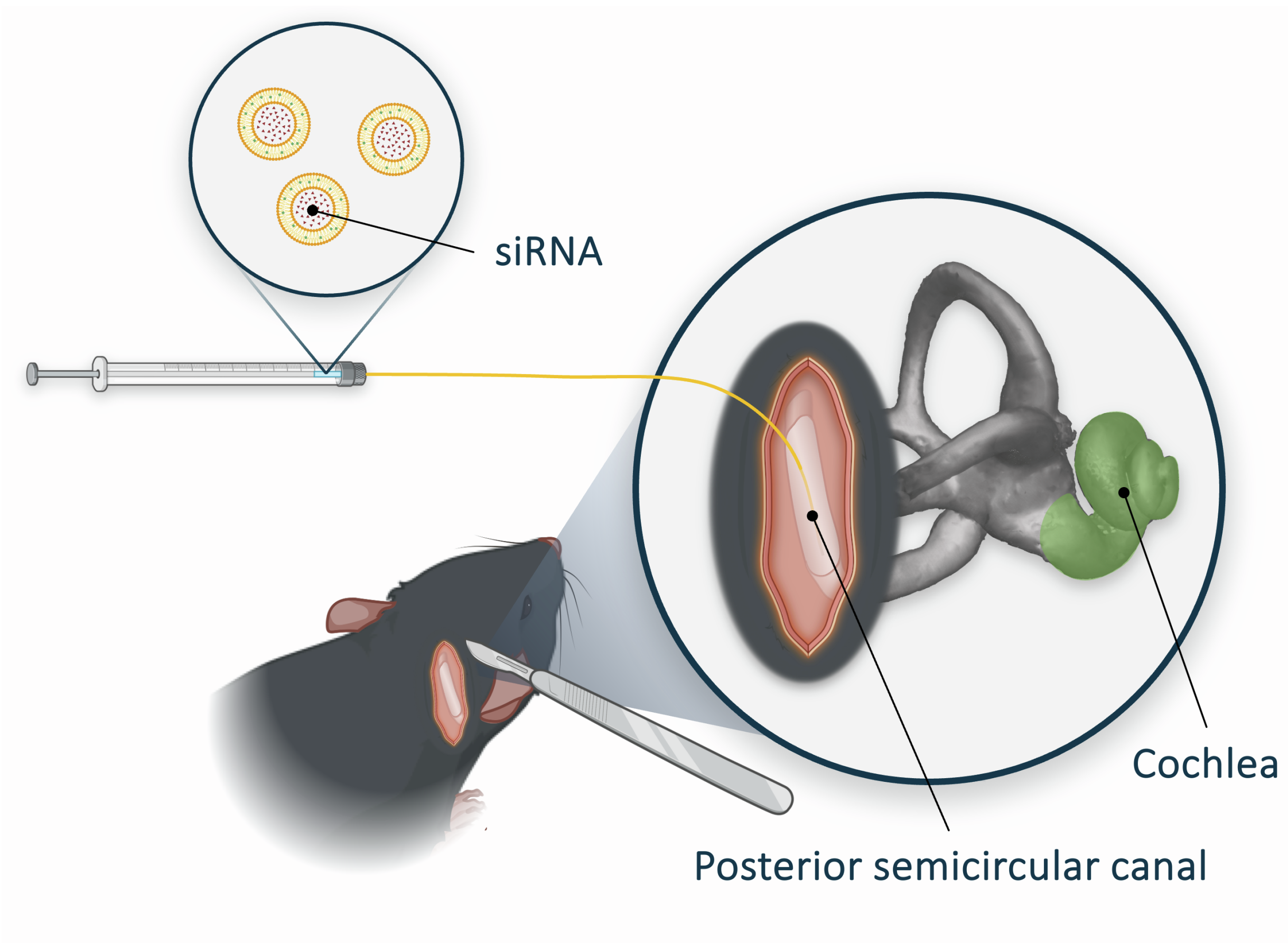

Figure S6 Schematic of siRNA delivery into the inner ear via the posterior semicircular canal. siRNA targeting *Nox3* or scrambled control siRNA was delivered into the inner ear 48 hours prior to stereotactic radiosurgery. Mice were anesthetized and placed in lateral position. Under surgical microscopy, a retroauricular incision was made, exposing the dorsal portion of the cervical trapezius muscle, located above the great auricular nerve. Following soft tissue dissection, the posterior semicircular canal was identified on the skull surface, carefully fenestrated using a 25G needle, and then cannulated. A total of 1 µL of siRNA solution was manually injected over 2 minutes through a microtube, and the injection site was sealed to prevent leakage. The wound was then closed and disinfected, and mice received local analgesia. Graphic elements adapted from BioRender.com.
