# Supplementary material for: NADPH oxidase 3 inhibition preserves hearing in mice after stereotactic radiosurgery
Source: Mol Ther Nucleic Acids. 2026 Jul 22;37(3):103036. doi: 10.1016/j.omtn.2026.103036 (PMC13487719; doi:10.1016/j.omtn.2026.103036)
Supplement: Supplementary file 2 [file mmc2.pdf]

Molecular Therapy
Nucleic Acids
Original Article

NADPH oxidase 3 inhibition preserves hearing in mice after stereotactic radiosurgery

Dimitrios Daskalou,[1,2] Francis Rousset,[1] Stéphanie Sgroi,[1] Lucie Oberhauser,[1] Jean-Philippe Thiran,[3,4] Constantin Tuleasca,[5,7] Ileana O. Jelescu,[4] Marc Levivier,[5,6] and Pascal Senn[1,2]

[1]The Inner Ear and Olfaction Lab, University of Geneva, Faculty of Medicine, 1211 Geneva, Switzerland; [2]Service of Otorhinolaryngology-Head and Neck Surgery, Department of Clinical Neurosciences, Geneva University Hospitals, 1205 Geneva, Switzerland; [3]Signal Processing Laboratory 5, École Polytechnique Fédérale de Lausanne (EPFL), 1015 Lausanne, Switzerland; [4]Department of Radiology, Lausanne University Hospital (CHUV), 1011 Lausanne, Switzerland; [5]Neurosurgery Service and Gamma Knife Center, Lausanne University Hospital (CHUV), Rue du Bugnon 44-46, BH-08, 1011 Lausanne, Switzerland; [6]Hôpital de La Tour, Centre NeuroKnife, Av. J.-D.-Maillard 3, 1217 Meyrin, Switzerland

Stereotactic radiosurgery (SRS) is a standard treatment for vestibular schwannoma but is associated with a substantial risk of progressive and irreversible hearing loss. NADPH oxidase 3 (NOX3), an enzyme predominantly expressed in the inner ear, has been identified as a key source of oxidative stress underlying cochlear injury. Using a mouse model of SRS-induced hearing loss, we found that genetic deletion of *Nox3* preserved auditory function and protected cochlear structures, including hair cells, synaptic ribbons, and spiral ganglion neurons. Complementary experiments using local delivery of a small interfering RNA directed against *Nox3* confirmed that transient inhibition markedly reduced hearing loss and tissue damage compared with scrambled controls. In addition, *Nox3* inhibition attenuated radiation-induced lipid peroxidation and macrophage infiltration within the cochlea. These findings identify NOX3-derived oxidative stress as an important upstream mediator of SRS-induced ototoxicity and demonstrate that its inhibition confers significant functional and structural protection. Given the predictable timing of SRS in clinical settings, a NOX3-targeted intervention may offer a feasible and selective strategy to prevent hearing loss as an adjunct to tumor-directed treatment.

INTRODUCTION

Vestibular schwannomas (VSs) are benign tumors of Schwann cell origin that arise mainly along the vestibular branch of the eighth cranial nerve. They are the most common neoplasms of the cerebellopontine angle in adults, with a lifetime prevalence exceeding 1 in 500 individuals.[1,2] A key treatment for small-to-medium VS is stereotactic radiosurgery (SRS), a targeted radiation technique.[3] While SRS achieves excellent tumor control, it does not prevent progressive hearing loss, a major long-term challenge, as over 40% of patients lose serviceable hearing within 4–6 years and up to 80% by 10 years post-treatment.[4,5] Even modest declines in hearing can substantially impair communication and quality of life in affected patients.[6]

Radiation-induced hearing loss is associated with structural damage to the mechanosensitive hair cells, auditory neurons, and stria vascularis within the cochlea.[7–9] Ionizing radiation causes cellular damage through both direct and indirect mechanisms. While direct DNA damage accounts for part of the response, most biological effects result from reactive oxygen species (ROS), leading to oxidative injury to DNA, proteins, and lipids.[10,11] A major enzymatic source of ROS is the NADPH oxidase (NOX) family.[12] Unlike mitochondria, which generate ROS as a metabolic byproduct, NOX enzymes produce ROS in a regulated manner. Among them, NOX3 is predominantly expressed in the inner ear and has been implicated in cochlear vulnerability to oxidative stress. In mice, *Nox3* inhibition has been shown to confer protection against noise-, cisplatin-, and age-related hearing loss.[13–15] Moreover, radiation has been shown to markedly increase NOX3 levels in the cochlea, as demonstrated in a rat model.[16] These findings suggest that NOX3 may play a key role in radiation-induced cochlear injury, yet its specific contribution remains unexplored.

We have previously established a mouse model of SRS-induced hearing loss that closely replicates the clinical pattern observed in VS patients.[17] We also developed an atraumatic small interfering RNA (siRNA) delivery method capable of achieving ≈70% *Nox3* silencing *in vivo*.[18] Building on this work, here we tested whether inhibiting NOX3 in mice could prevent cochlear damage following SRS. Using both *Nox3*-deficient mice and *in vivo* delivery of siRNA targeting *Nox3*, we assessed auditory function and cochlear integrity after unilateral irradiation. In addition, we investigated whether NOX3 inhibition modulates radiation-induced oxidative stress and the associated inflammatory response within the cochlea. NOX3 inhibition attenuated both the functional and histological effects of radiation, reduced lipid peroxidation, and limited macrophage infiltration,

Received 29 December 2025; accepted 21 July 2026;
https://doi.org/10.1016/j.omtn.2026.103036.

[7]Present addresses: Clinique de La Source, 1004 Lausanne, Switzerland; Swiss Medical Network, 1006 Lausanne, Switzerland; University of Medicine and Pharmacy “Grigore T. Popa,” 700115 Iaşi, Romania

Correspondence: Dimitrios Daskalou, The Inner Ear and Olfaction Lab, University of Geneva, Faculty of Medicine, 1211 Geneva, Switzerland.
E-mail: dimitrios.daskalou@hug.ch

Published by Elsevier Inc. on behalf of The American Society of Gene and Cell Therapy.
This is an open access article under the CC BY license (http://creativecommons.org/licenses/by/4.0/).

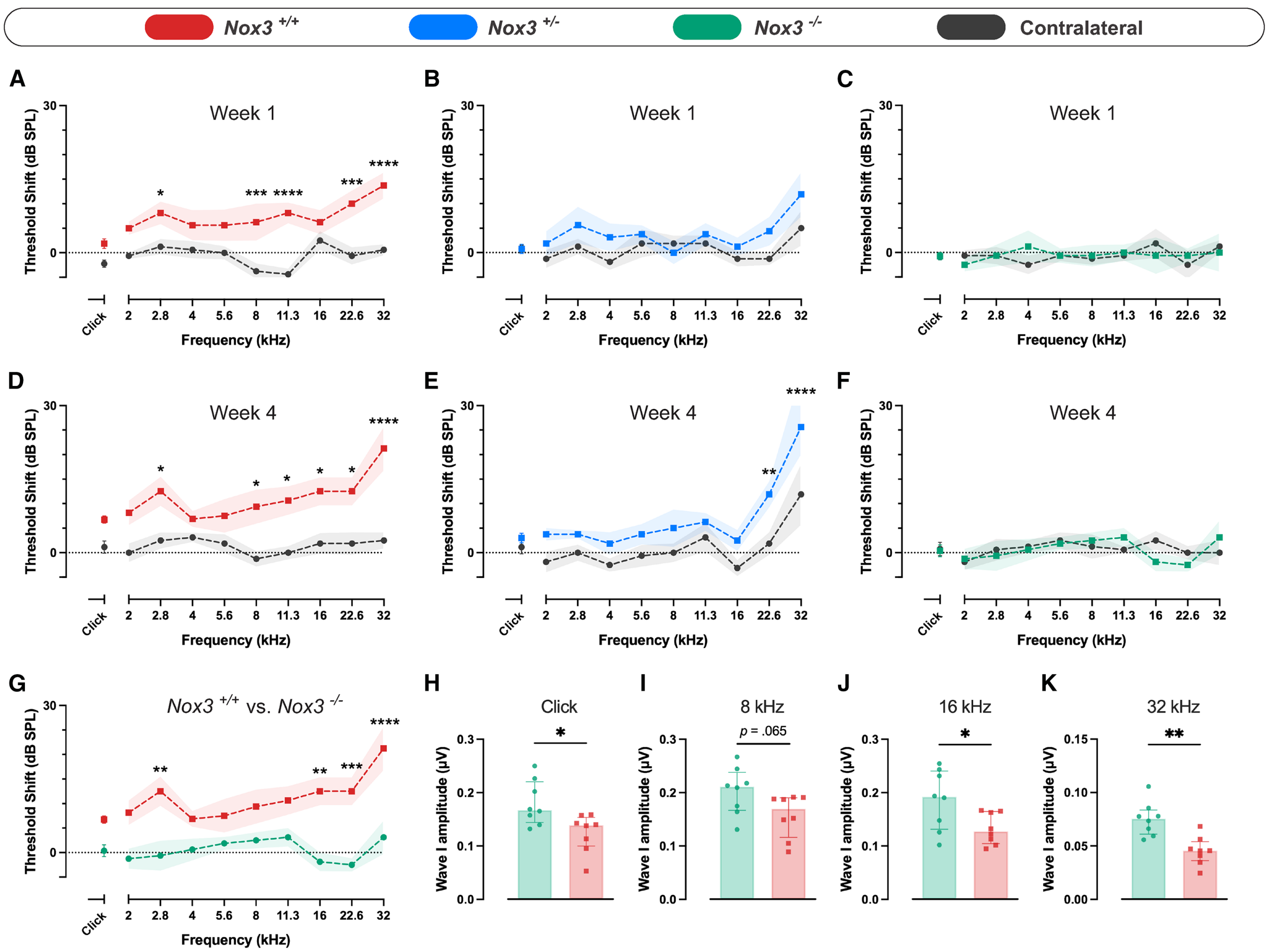

Figure 1. Auditory brainstem response threshold shifts and wave I amplitude changes following unilateral cochlear irradiation in Nox3 genotype groups

(A–C) Auditory brainstem response (ABR) threshold shifts at week 1 post-irradiation in wild-type (*Nox3*$^{+/+}$; $n = 8$), heterozygous (*Nox3*$^{+/-}$; $n = 8$), and *Nox3*-deficient (*Nox3*$^{-/-}$; $n = 8$) mice. Interaural differences were evident only in *Nox3*$^{+/+}$ mice, with the largest separation at 32 kHz. (D–F) Mean ABR threshold shifts at week 4 post-irradiation in the same groups (each $n = 8$). Threshold elevations progressed in *Nox3*$^{+/+}$ mice, were less pronounced in *Nox3*$^{+/-}$ mice, and were absent in *Nox3*$^{-/-}$ mice. (G) Direct comparison of ABR threshold shifts from irradiated ears between *Nox3*$^{+/+}$ and *Nox3*$^{-/-}$ mice at week 4. Threshold shifts were significantly higher in *Nox3*$^{+/+}$ mice at multiple frequencies. (H–K) Wave I amplitudes at week 4 from click stimulation (H) and tone bursts at 8 kHz (I), 16 kHz (J), and 32 kHz (K). Irradiated ears of *Nox3*$^{+/+}$ mice displayed reduced amplitudes compared with *Nox3*$^{-/-}$ mice. Data are presented as means with shaded areas representing SEM for ABR threshold shifts (A–G). Data are represented as medians with IQR for amplitudes (H–K). Asterisks indicate statistical significance (*$p < 0.05$, **$p < 0.01$, ***$p < 0.001$, and ****$p < 0.0001$). Statistical analysis was performed using two-way repeated measures ANOVA with Bonferroni correction for *post hoc* pairwise comparisons for threshold shifts (A–G) and Mann-Whitney test for amplitude comparisons (H–K).

supporting its role as a key contributor to cochlear injury and identifying it as a promising therapeutic target.

RESULTS

Nox3 deficiency preserves ABR thresholds following unilateral cochlear irradiation

At week 1 after SRS, wild-type mice (*Nox3*$^{+/+}$) began to show auditory brainstem response (ABR) threshold shifts, with the most significant shift being at 32 kHz compared with the contralateral ear (mean difference: 13.1 dB; $p < 0.0001$; 95% confidence interval [CI]: 6.5–19.7; Figure 1A). At this time point, neither heterozygous (*Nox3*$^{+/-}$) nor *Nox3*-deficient (*Nox3*$^{-/-}$) mice presented significant interaural differences (Figures 1B and 1C). At week 4, hearing loss progressed in wild-type mice, especially in the high frequencies, with a mean interaural difference of 18.8 dB at 32 kHz ($p < 0.0001$; 95% CI: 9.2–28.3; Figure 1D). A less pronounced effect was observed in the heterozygous group with a mean difference between irradiated and contralateral ears of 13.8 dB at 32 kHz ($p < 0.0001$; 95% CI: 6.7–20.8; Figure 1E). Interestingly, no hearing loss was detected in the *Nox3*-deficient group across all measured frequencies up to 32 kHz (Figure 1F). At week 4 post-irradiation, direct comparison of the irradiated ears between wild-type and *Nox3*-deficient mice revealed

significant threshold shift differences at 2.8, 16, 22.6, and 32 kHz, with the largest difference observed at 32 kHz (mean difference: 18.1 dB; $p < 0.0001$; 95% CI: 7.8–28.4; Figure 1G).

We analyzed the amplitudes of waves I, III, and IV after suprathreshold stimulation and compared the median values of the irradiated ears between wild-type and *Nox3*-deficient mice at week 4 after SRS. The median wave I amplitude was lower in wild-type mice than in *Nox3*-deficient mice after click stimulation (0.14 vs. 0.17 μV; $p = 0.02$) and after pure tone stimulation at 16 kHz (0.13 vs. 0.19 μV; $p = 0.049$) and 32 kHz (0.05 vs. 0.08 μV; $p = 0.002$; Figures 1H–1K). For wave III, amplitudes were also lower in wild-type mice after click stimulation (0.14 vs. 0.17 μV; $p = 0.02$) and at 32 kHz (0.07 vs. 0.11 μV; $p = 0.03$; Figures S1A and S1D). No differences in wave III amplitudes were observed at 8 and 16 kHz (Figures S1B and S1C), and wave IV amplitudes did not differ between the two groups (Figures S1E–S1H).

In vivo siRNA-mediated inhibition of Nox3 attenuates radiation-induced hearing loss

We investigated whether *in vivo* inhibition of *Nox3* could preserve hearing following irradiation. To this end, an siRNA targeting *Nox3* mRNA (siNox3) was delivered via the posterior semicircular canal (PSCC) approach 48 h prior to SRS. ABR threshold shifts at weeks 1 and 4 were compared with those in a control group treated with scrambled siRNA (siScr) and irradiated using the same protocol. At week 1, siScr-treated mice exhibited significant threshold shifts in the irradiated ear compared with the contralateral side across all measured frequencies, with average interaural differences ranging from 6 to 12 dB (Figure 2A). In contrast, no threshold shift was detected in siNox3-treated mice at this time point (Figure 2B). By week 4, hearing loss had progressed in the siScr group, particularly at high frequencies, with a mean interaural difference of 21.1 dB at 22.6 kHz ($p < 0.0001$; 95% CI: 15.1–27) and 25.7 dB at 32 kHz ($p < 0.0001$; 95% CI: 19.8–31.7; Figure 2C). A significantly milder effect was observed in the siNox3 group, with mean interaural differences of 5 dB at 22.6 kHz ($p = 0.08$; 95% CI: −0.3 to 10.3) and 7.5 dB at 32 kHz ($p = 0.008$; 95% CI: 2.3–12.8; Figure 2D).

Direct comparison of irradiated ears at week 4 showed significantly greater threshold shifts in the siScr group than in the siNox3 group at 5.6, 11.3, 16, 22.6, and 32 kHz, with the largest difference at 32 kHz (mean difference: 17.5 dB; $p < 0.0001$; 95% CI: 7.3–27.7; Figure 2E). In line with the observed difference in threshold shifts at 32 kHz at week 4, we found lower median amplitudes in the siScr group compared with the siNox3 group for both wave I (0.03 μV vs. 0.05 μV; $p = 0.03$) and wave III (0.05 μV vs. 0.07 μV; $p = 0.04$; Figures 2F and 2G). No other differences in the amplitudes of waves I, III, and IV were observed after click stimulation or at 8 and 16 kHz (Figures S2A–S2J).

Nox3 inhibition mitigates radiation-induced outer hair cell loss

We studied the viability of hair cells in cochleae at week 4 after SRS. Inner hair cells (IHCs) were preserved across the three experimental groups of the *Nox3* genotype study, except for a minor IHC loss at 45.2 kHz detected in the wild-type group (Figures S3A–S3C). In contrast, significant outer hair cell (OHC) loss was observed, particularly in high-frequency cochlear regions. In wild-type mice, OHCs were reduced in the irradiated cochlea compared with the contralateral side by 6 percentage points at 16 kHz ($p = 0.005$; 95% CI: 1.5–10.4), 7.6 at 32 kHz ($p = 0.0004$; 95% CI: 3.1–12), and 22.1 at 45.2 kHz ($p < 0.0001$; 95% CI: 17.7–26.6; Figures 3A and 3D). In the heterozygous group, a significant interaural difference of 14 percentage points was detected at 45.2 kHz ($p = 0.0007$; 95% CI: 6.9–21; Figures 3B and 3D). In the *Nox3*-deficient group, the interaural difference at 45.2 kHz was 8.9 percentage points ($p < 0.0001$; 95% CI: 6.4–11.4; Figures 3C and 3D).

IHCs were preserved in both siScr- and siNox3-treated mice, except for a minor IHC loss at 32 kHz in the siScr group (Figures S4A and S4B). Similar to wild-type mice, siScr-treated mice exhibited OHC loss in the irradiated cochlea, with an average interaural difference of 18.3 percentage points at the 45.2 kHz region ($p < 0.0001$; 95% CI: 11.1–25.5; Figures 4A and 4C). At the same frequency, the siNox3 group showed a smaller interaural difference of 9.8 percentage points ($p = 0.0009$; 95% CI: 3.8–15.8; Figures 4B and 4C).

Nox3 inhibition preserves CtBP2-positive presynaptic ribbons

To assess presynaptic ribbon integrity, we quantified CtBP2-positive puncta in inner hair cells at week 4 after SRS. In wild-type mice of the *Nox3* genotype study, CtBP2-positive ribbon counts were mildly reduced in the 8–32 kHz cochlear regions compared with the contralateral side. At 45.2 kHz, the interaural difference was more pronounced, with an average reduction of 5 ribbons per IHC, reaching statistical significance ($p = 0.0005$; 95% CI: 2.1–7.8; Figures 3E and 3H). In heterozygous mice, a minor but non-significant reduction in ribbon counts was observed at high frequencies (Figures 3F and 3H). In contrast, ribbon counts were identical between irradiated and contralateral cochleae across all measured frequencies in *Nox3*-deficient mice (Figures 3G and 3H).

In siScr- and siNox3-treated mice, CtBP2-positive ribbon loss patterns mirrored those observed in wild-type and *Nox3*-deficient groups, respectively. In the siScr group, compared with the contralateral side, ribbon counts were reduced by 2 ribbons per IHC at 22.6 kHz ($p = 0.009$; 95% CI: 0.4–3.8), by 1.8 at 32 kHz ($p = 0.03$; 95% CI: 0.1–3.5), and by 2.4 at 45.2 kHz ($p = 0.003$; 95% CI: 0.7–4.0; Figures 4D and 4F). In contrast, no interaural differences in ribbon counts were detected in siNox3-treated mice (Figures 4E and 4F).

Nox3 inhibition preserves spiral ganglion neuron density

We assessed spiral ganglion neuron (SGN) density at week 4 after SRS. In wild-type mice of the *Nox3* genotype study, SGN density in the irradiated cochlea was reduced compared with the contralateral side by 637 SGN/mm^2 in the middle turn ($p = 0.002$; 95% CI: 372.5–901.5) and by 414 SGN/mm^2 in the basal turn ($p = 0.01$; 95% CI: 149.4–678.5; Figures 3I and 3L). In contrast, no interaural

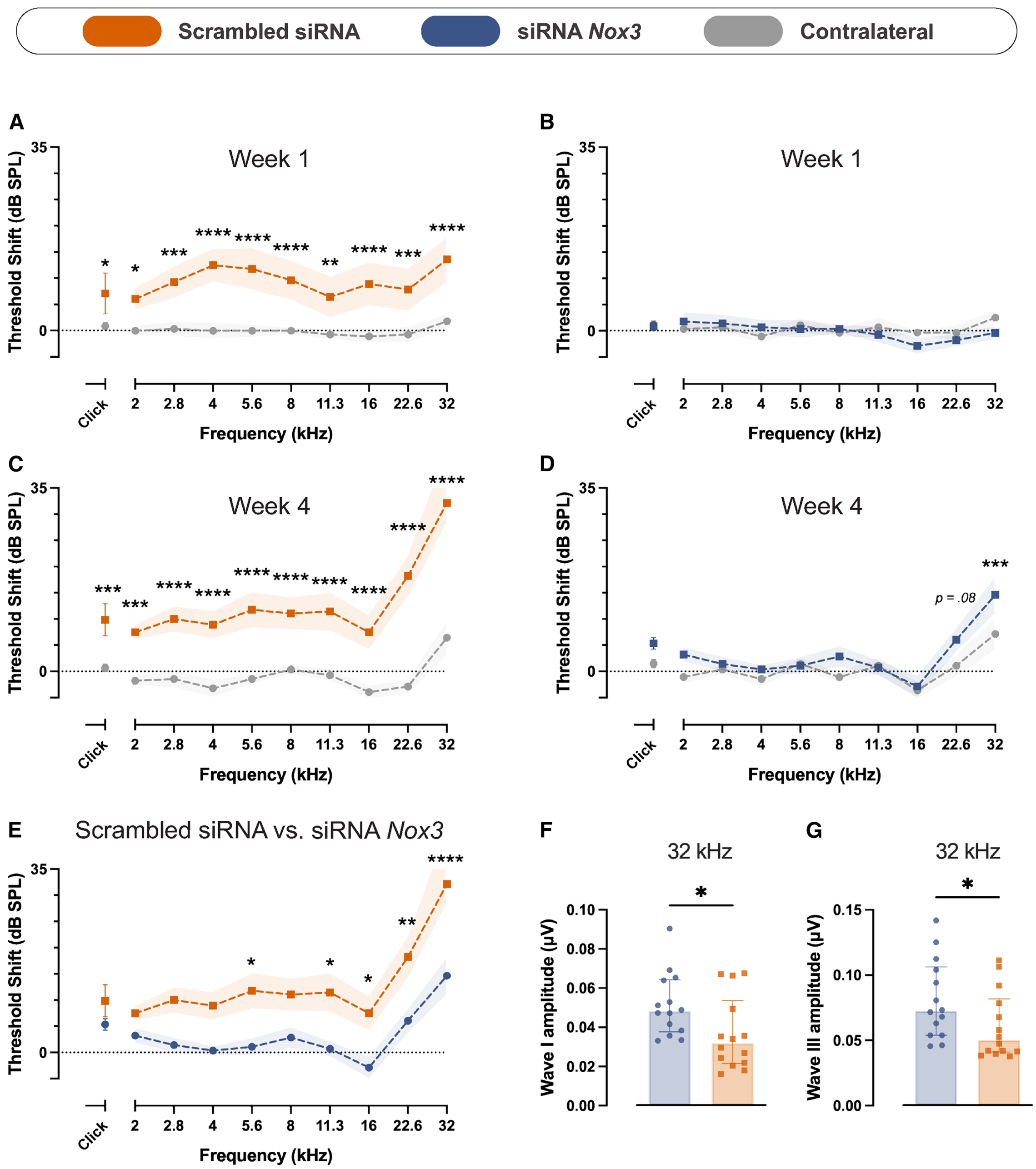

Figure 2. Auditory brainstem response threshold shifts and wave amplitudes following siRNA-mediated Nox3 inhibition prior to irradiation

(A and B) Auditory brainstem response (ABR) threshold shifts at week 1 post-irradiation in mice treated with scrambled control siRNA (siScr; A) or siRNA targeting *Nox3* (siNox3; B) via the posterior semicircular canal 48 h before irradiation (each $n = 14$). Significant interaural differences were observed in siScr-treated mice, whereas siNox3-treated mice showed no measurable threshold shifts. (C and D) Threshold shifts at week 4 in the same groups. Hearing loss progressed in siScr-treated mice, particularly at high frequencies, while the siNox3 group showed attenuated threshold elevations. (E) Direct comparison of ABR threshold shifts from irradiated ears between siScr- and siNox3-treated mice at week 4. Threshold shifts were significantly higher in the siScr group at multiple frequencies. (F and G) ABR wave I (F) and wave III (G) amplitudes after suprathreshold stimulation at 32 kHz, measured at week 4. Both wave I and III amplitudes were significantly reduced in the siScr group compared with siNox3-treated mice. Data are presented as means with shaded SEM for threshold shifts (A–E). Data are represented as medians with IQR for amplitudes (F and G). Asterisks denote statistical significance (*$p < 0.05$, **$p < 0.01$, ***$p < 0.001$, and ****$p < 0.0001$). Threshold shifts were analyzed using two-way repeated measures ANOVA with Bonferroni correction; wave amplitudes were compared using Mann-Whitney tests.

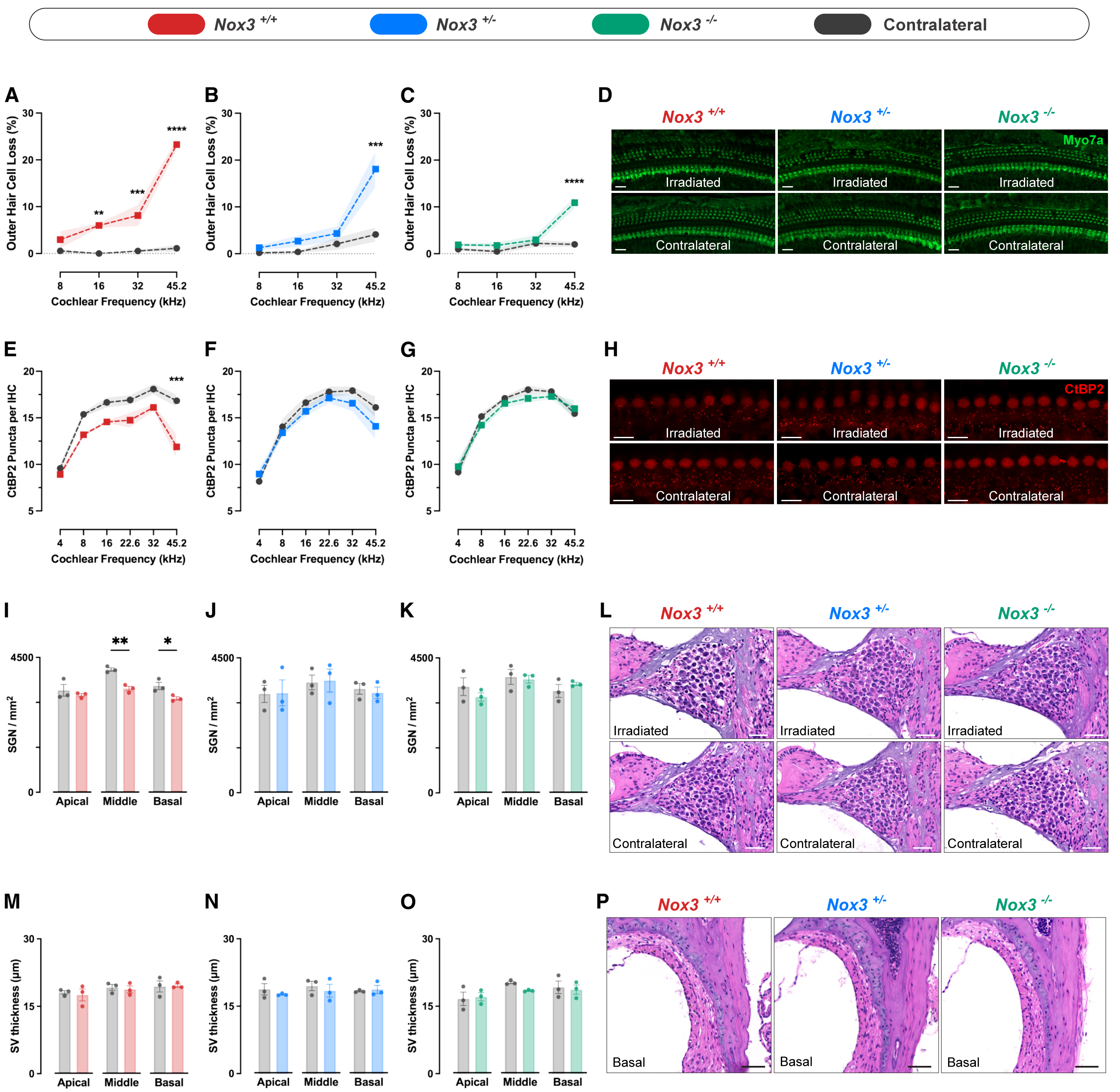

Figure 3. Cochlear histopathology at 4 weeks post-irradiation in Nox3 genotype groups

(A–C) Percentage outer hair cell (OHC) loss across cochlear frequencies in wild-type (*Nox3*$^{+/+}$; A), heterozygous (*Nox3*$^{+/-}$; B), and *Nox3*-deficient (*Nox3*$^{-/-}$; C) mice (each *n* = 4), relative to the contralateral cochlea. OHC loss was most pronounced in the high-frequency regions of *Nox3*$^{+/+}$ mice and minimal in *Nox3*$^{-/-}$ animals. (D) Representative Myo7a-stained cochlear segments at the 45.2 kHz region in irradiated and contralateral cochleae from *Nox3*$^{+/+}$, *Nox3*$^{+/-}$, and *Nox3*$^{-/-}$ mice. Scale bars, 20 μm. (E–G) Ribbon density measured as CtBP2-positive puncta per inner hair cell (IHC) across cochlear frequencies in *Nox3*$^{+/+}$ (E), *Nox3*$^{+/-}$ (F), and *Nox3*$^{-/-}$ (G) mice (each *n* = 4). A significant reduction was observed in *Nox3*$^{+/+}$ mice at the 45.2 kHz region.

(H) Representative CtBP2-stained IHCs from irradiated and contralateral cochleae of *Nox3*$^{+/+}$, *Nox3*$^{+/-}$, and *Nox3*$^{-/-}$ mice at the 45.2 kHz region. Scale bars, 10 μm. (I–K) Spiral ganglion neuron (SGN) density in basal, middle, and apical turns in *Nox3*$^{+/+}$ (I), *Nox3*$^{+/-}$ (J), and *Nox3*$^{-/-}$ (K) mice (*n* = 3 per group). SGN loss was evident in the basal and middle turns of *Nox3*$^{+/+}$ cochleae but absent in *Nox3*$^{+/-}$and *Nox3*$^{-/-}$ groups. (L) Representative hematoxylin and eosin-stained mid-modiolar cochlear sections illustrating SGN density differences between irradiated and contralateral ears for the middle turn in *Nox3*$^{+/+}$, *Nox3*$^{+/-}$, and *Nox3*$^{-/-}$ mice. Scale bars, 40 μm. (M–O) Stria

(legend continued on next page)

difference in SGN density was detected in either the heterozygous or the *Nox3*-deficient group (Figures 3J–3L).

In siScr-treated mice, SGN density was reduced by 372.5 SGN/mm^2 in the middle turn ($p = 0.01$; 95% CI: 100–645) and by 409 SGN/mm^2 in the basal turn compared with the contralateral cochlea ($p = 0.006$; 95% CI: 136.8–681.8; Figures 4G and 4I). No significant interaural difference was observed in siNox3-treated mice (Figures 4H and 4I).

Stria vascularis thickness remains unchanged across experimental groups

We studied stria vascularis thickness by comparing averaged measurements per cochlear turn in the three experimental groups of the *Nox3* genotype study and in the siRNA study. No significant differences were detected among wild-type, heterozygous, and *Nox3*-deficient mice, nor between siScr- and siNox3-treated mice (Figures 3M–3P and 4J–4L).

Nox3 inhibition reduces radiation-induced oxidative stress and macrophage infiltration

We assessed oxidative stress and macrophage response in cochlear cryosections at week 4 after SRS using 4-hydroxynonenal (4-HNE) and Iba1 immunostaining, respectively. In siScr-treated mice, 4-HNE immunoreactivity was increased in the irradiated cochlea compared with the contralateral side in the organ of Corti. The percentage of 4-HNE-positive area was higher in the irradiated cochlea by 16.3 percentage points in the middle turn ($p = 0.02$; 95% CI: 3.9–28.6) and by 19.3 percentage points in the basal turn ($p = 0.007$; 95% CI: 7–31.7), while no significant difference was observed in the apical turn (Figures 5A and 5C). In the stria vascularis, the percentage of 4-HNE-positive area was increased in the irradiated cochlea by 18.3 percentage points in the basal turn ($p = 0.005$; 95% CI: 7.1–29.5), whereas no significant interaural differences were detected in the apical and middle turns (Figures 5D and 5F). In contrast, siNox3-treated mice did not show significant interaural differences in percentage 4-HNE-positive area in either the organ of Corti or the stria vascularis across all cochlear turns (Figures 5B, 5C, 5E, and 5F).

We next evaluated macrophage infiltration using Iba1 immunostaining. In siScr-treated mice, Iba1-positive cell density in SGNs was increased in the irradiated cochlea by 109.5 cells/mm^2 in the apical turn ($p = 0.006$; 95% CI: 39.9–179.1), by 126.3 cells/mm^2 in the middle turn ($p = 0.003$; 95% CI: 56.7–195.9), and by 99.6 cells/mm^2 in the basal turn ($p = 0.009$; 95% CI: 30–169.2; Figures 5G and 5I). A similar increase was observed in the spiral ligament, where Iba1-positive cell density was higher in the irradiated cochlea by 224.1 cells/mm^2 in the apical turn ($p = 0.0003$; 95% CI: 141–307.2), by 127.4 cells/mm^2 in the middle turn ($p = 0.007$; 95% CI: 44.3–210.6), and by 163.4 cells/mm^2 in the basal turn ($p = 0.002$; 95% CI: 80.3–246.5; Figures 5J and 5L). In addition, Iba1-positive cells were frequently observed clustered in the lower portion of the spiral ligament in irradiated cochleae (Figure 5L). In contrast, siNox3-treated mice showed no significant interaural differences in Iba1-positive cell density in SGNs across all cochlear turns (Figures 5H and 5I). In the spiral ligament, Iba1-positive cell density was higher in the irradiated cochlea by 110.4 cells/mm^2 in the apical turn ($p = 0.04$; 95% CI: 8–212.8) and by 107.2 cells/mm^2 in the middle turn ($p = 0.04$; 95% CI: 4.8–209.7), whereas no significant difference was observed in the basal turn (Figures 5K and 5L).

Additional analyses revealed no significant interaural differences in percentage 4-HNE-positive area in SGNs in either siScr- or siNox3-treated mice across all cochlear turns (Figures S5A and S5B). Similarly, no significant interaural differences in Iba1-positive cell density were observed in the stria vascularis in either group (Figures S5C and S5D).

DISCUSSION

In this study, we provide evidence that implicates NOX3 in the development of radiation-induced cochlear injury. Using both genetic deletion and targeted siRNA delivery, we show that inhibition of *Nox3* mitigates auditory dysfunction and cochlear pathology following SRS. *Nox3*-deficient mice showed preserved auditory thresholds and were largely protected from radiation-induced OHC loss, synaptic ribbon loss, and SGN degeneration. Similarly, *in vivo* delivery of siRNA targeting *Nox3*, 48 h before SRS, substantially attenuated functional decline and histological damage compared with scrambled controls, and reduced markers of oxidative stress and macrophage infiltration within the cochlea.

We observed that both wild-type and siScr-treated mice developed SRS-induced cochlear injury affecting OHCs, synaptic ribbons, and SGNs. Histopathological studies in both humans and animal models have similarly shown that irradiation can damage multiple cochlear structures, leading to progressive hearing loss even in the absence of a VS.[7–9,19–23] These structural changes result from a combination of direct and indirect cellular injury. In this context, radiation-induced cochlear injury is a multifactorial process involving both direct DNA damage and indirect mechanisms mediated by ROS. While ionizing radiation can damage DNA directly, the majority of injury arises indirectly through the generation of ROS.[10,11] These highly reactive molecules not only cause oxidative damage to DNA, proteins, and lipids but also trigger mitochondrial dysfunction, cytochrome *c* release, and activation of stress-related signaling pathways such as p53, p38 mitogen-activated protein kinase, and

vascularis (SV) thickness in *Nox3*$^{+/+}$ (M), *Nox3*$^{+/-}$ (N), and *Nox3*$^{-/-}$ (O) mice ($n = 3$ per group), showing no significant differences between irradiated and contralateral cochleae. (P) Representative histological sections showing SV morphology in the basal turn of irradiated cochleae from *Nox3*$^{+/+}$, *Nox3*$^{+/-}$, and *Nox3*$^{-/-}$ mice. Scale bars, 50 μm. Data are presented as means with shaded areas or error bars representing SEM. Asterisks indicate statistical significance (*$p < 0.05$, **$p < 0.01$, ***$p < 0.001$, and ****$p < 0.0001$). Statistical analysis was performed using two-way repeated measures ANOVA with Bonferroni correction for post hoc pairwise comparisons.

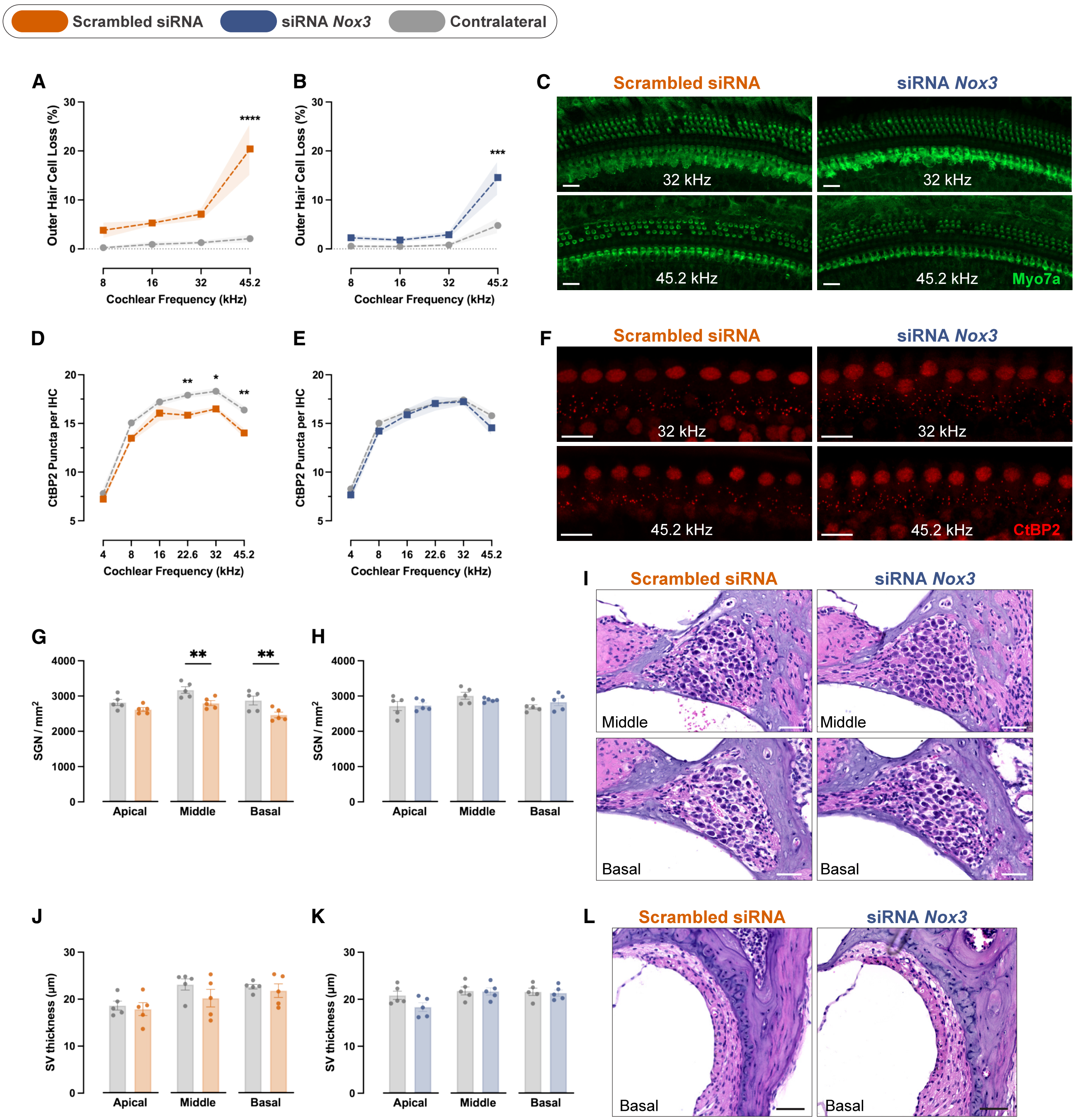

Figure 4. Cochlear histopathology at 4 weeks post-irradiation in siRNA-treated mice

(A and B) Percentage outer hair cell (OHC) loss across cochlear frequencies in scrambled control siRNA (siScr; A; *n* = 7) and siRNA targeting *Nox3* (siNox3; B; *n* = 8) groups, relative to the contralateral cochlea. OHC loss was most pronounced in the high-frequency regions of siScr-treated mice and attenuated in siNox3-treated animals. (C) Representative Myo7a-stained cochlear segments at 32 and 45.2 kHz regions of irradiated cochleae from siScr- and siNox3-treated mice. Scale bars, 20 μm. (D and E) Ribbon density measured as CtBP2-positive puncta per inner hair cell (IHC) across cochlear frequencies in siScr (D; *n* = 7) and siNox3 (E; *n* = 8) groups. Ribbon loss was detected in the high-frequency regions of siScr-treated mice but not in siNox3-treated animals. (F) Representative CtBP2-stained IHCs of irradiated cochleae of siScr- and siNox3-treated mice at 32 and 45.2 kHz regions. Scale bars, 10 μm. (G and H) Spiral ganglion neuron (SGN) density in basal, middle, and apical turns of siScr (G) and siNox3 (H) groups (*n* = 5 per group). SGN loss was observed in the basal and middle turns of siScr-treated cochleae, but not in siNox3-treated mice. (I) Representative hematoxylin and eosin-stained mid-modiolar cochlear sections of irradiated cochleae illustrating SGN density differences between siScr- and siNox3-treated mice for the basal and

(legend continued on next page)

c-Jun N-terminal kinase.[11,24–28] Beyond direct cytotoxicity, ROS act as secondary messengers capable of inducing epigenetic modifications and transcriptional changes, which can disturb calcium and glutamate homeostasis and promote excitotoxic signaling in SGNs.[14,29–32] In parallel, pro-inflammatory mediators including tumor necrosis factor-alpha, interleukin-1β, interleukin-6, and C-C motif chemokine ligand 2 have been reported to be upregulated in the irradiated cochlea, further disrupting cochlear homeostasis.[33] In line with these mechanisms, we observed increased lipid peroxidation and macrophage infiltration in the irradiated cochlea, as evidenced by increased 4-HNE immunoreactivity and Iba1-positive cell density in siScr-treated mice. These changes were significantly attenuated by *Nox3* inhibition, indicating that NOX3-derived ROS contributes not only to direct cellular injury but also to the establishment of a pro-inflammatory microenvironment within the cochlea. Taken together, this persistent oxidative and inflammatory environment is thought to underlie the delayed and progressive nature of radiation-induced hearing loss, consistent with both clinical observations and the temporal evolution of hearing thresholds in our experimental model.[10]

Most preclinical attempts at cochlear radioprotection have therefore targeted downstream oxidative or inflammatory cascades using pharmacological strategies. These include antioxidant, redox-modulating, or cytoprotective agents such as N-acetylcysteine, melatonin, epicatechin, L-carnitine, metformin, tetramethylpyrazine, and amifostine, as well as anti-inflammatory approaches including NF-κB inhibition with JSH-23 and dexamethasone-based strategies.[9,19,20,23,34–39] Although their mechanisms differ, these interventions generally act downstream of the initial radiation insult by reducing oxidative injury, limiting inflammatory signaling, supporting mitochondrial function, improving microcirculatory homeostasis, or providing broader cytoprotection. Delivery routes have also differed across these studies, ranging from systemic administration to intratympanic delivery, whereas the NOX3-directed siRNA strategy evaluated here relies on direct local inner-ear administration. Several have shown protective effects in small animal studies; however, efficacy has been variable, as metformin did not show significant radioprotection *in vitro* or *in vivo*, and intratympanic dexamethasone alone did not significantly improve radiation-induced hearing thresholds.[19,38] Moreover, these approaches have not been clinically tested for preventing hearing loss after SRS, and their efficacy in this specific setting remains to be demonstrated. A key limitation of broadly acting antioxidant or anti-inflammatory strategies is that they mainly counteract ROS or inflammation after activation of the injury cascade, rather than preventing sustained ROS production at the source. Ionizing radiation has been shown to increase ROS levels rapidly, within the first hour after exposure, indicating that effective protection requires intervention at the point of ROS generation.[40] In this regard, NOX3 represents a particularly relevant target. Its expression has been shown to increase as early as one hour after irradiation *in vitro*.[41] *In vivo* rat models have further confirmed a marked rise in cochlear NOX3 levels after cranial irradiation.[16] Together, these findings indicate that NOX3 is upregulated precisely when and where injury begins. In the present study, we demonstrated that inhibition of *Nox3* effectively mitigated ABR threshold shifts and histological injury, thereby providing direct experimental support for NOX3 as a relevant therapeutic target within the broader ROS-mediated injury cascade. In addition, we show that NOX3 inhibition reduces both lipid peroxidation and macrophage infiltration, further supporting its role as an upstream driver of oxidative and inflammatory responses following irradiation. Another concern is that broad antioxidants or anti-inflammatory agents may compromise tumor control.[42–45] Oxidative stress is a key mediator of the cytotoxic effect of SRS in VS,[46] and generic scavengers administered even locally in the cochlea could reach the tumor through exchange between the perilymph and cerebrospinal fluid.[47,48] By contrast, NOX3 expression is largely restricted to the inner ear, with no clearly established essential physiological role in adult mammals, including humans.[14,49,50] Together, these considerations and our findings highlight NOX3 inhibition as a promising and potentially selective approach for cochlear radioprotection. Its local delivery and the predominant expression of NOX3 in the inner ear support its rationale as an otoprotective strategy administered alongside SRS, while future tumor-bearing studies will be required to directly assess its effect on tumor response.

In our study, we observed a higher vulnerability of the basal turn of the cochlea to irradiation, consistent with our previous study[17] and with the hearing loss pattern seen in patients after irradiation.[10,51] Moreover, this regional susceptibility mirrors hearing loss patterns seen in other etiologies such as ototoxic drugs, noise exposure, and aging.[15,52,53] Several mechanisms may account for this gradient of vulnerability. The basal turn exhibits greater oxidative stress responses, with upregulation of redox-related genes and lower levels of intrinsic antioxidants such as glutathione.[54,55] Importantly, our group and independent studies have previously demonstrated that NOX3 follows a tonotopic pattern of induced expression after noise trauma, with higher levels in the basal cochlea.[15,50] Given that radiation-induced injury is largely mediated by ROS, it is plausible that increased NOX3 induction, combined with reduced antioxidant defenses, underlies the basal turn's sensitivity. Consistent with this hypothesis, we observed that radiation-induced oxidative damage, assessed by 4-HNE immunoreactivity, was most pronounced in the basal turn of siScr-treated mice, particularly at the organ of Corti and the stria vascularis, further supporting a spatial gradient of oxidative stress along the cochlea. Thus, inhibition of NOX3 may

middle turns. Scale bars, 40 μm. (J and K) Stria vascularis (SV) thickness in siScr (J; $n = 5$) and siNox3 (K; $n = 5$) groups, showing no significant differences between irradiated and contralateral cochleae. (L) Representative histological sections showing SV morphology in the basal turn of irradiated cochleae from siScr- and siNox3-treated mice. Scale bars, 50 μm. Data are presented as means with shaded areas or error bars representing SEM. Asterisks indicate statistical significance (*$p < 0.05$, **$p < 0.01$, ***$p < 0.001$, and ****$p < 0.0001$). Statistical analysis was performed using two-way repeated measures ANOVA with Bonferroni correction for post hoc pairwise comparisons.

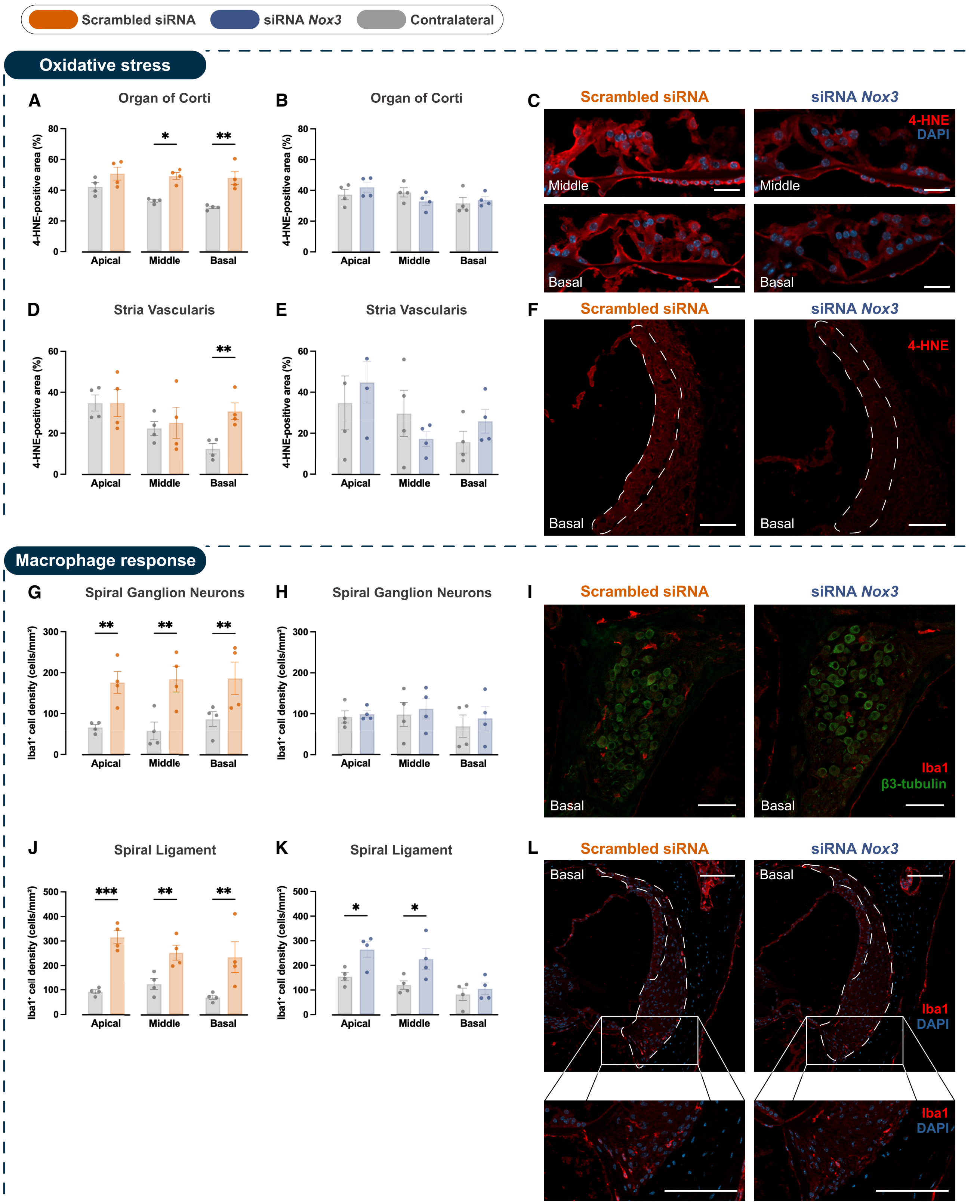

(legend on next page)

counteract the excessive basal-turn ROS burden, explaining the protection we observed in this study.

Although NOX3 inhibition markedly reduced radiation-induced cochlear injury, protection was not complete, and siRNA-treated animals showed a smaller benefit than *Nox3*-deficient mice, approaching the phenotype of heterozygous animals. Several factors likely explain this difference. First, siRNA-mediated knockdown is partial; our construct achieves $\approx 70\%$ silencing at 48 h after injection, leaving residual NOX3 expression that may be sufficient to sustain oxidative stress.[18] Second, siRNA molecules are subject to degradation over days, allowing recovery of NOX3 expression during the 4-week observation period.[56,57] More durable strategies, such as microRNA-based inhibition or stabilized siRNA formulations, may extend the window of protection.[58] In addition, the optimal therapeutic window for NOX3 inhibition remains to be defined. Future studies should therefore evaluate the efficacy of post-SRS administration and optimized delivery strategies enabling sustained *Nox3* suppression. Third, radiation injury may not be exclusively mediated by NOX3-derived ROS. Mitochondria are a well-established source of oxidative stress in other ototoxic settings, as impaired oxidative phosphorylation and cytochrome *c* release amplify ROS production and trigger apoptosis.[59,60] These effects are partially buffered by antioxidant defenses such as mitochondrial superoxide dismutase and glutathione peroxidase but may still contribute to residual damage.[61,62] In contrast, NOX3 is a regulated enzymatic source localized to cellular membranes, where it directly engages redox-sensitive signaling pathways, including excitotoxic glutamatergic signaling and pro-inflammatory cascades.[14,63] A plausible model is therefore that mitochondrial ROS establishes a basal oxidative load, while NOX3-derived ROS provides a spatially targeted burst that tips the balance toward excitotoxicity and degeneration. This raises the possibility that optimal radioprotection may require combined strategies. One approach could be to couple NOX3 inhibition with mitochondria-targeted antioxidants such as MitoQ, which has been shown to scavenge mitochondrial ROS effectively and protect against cochlear injury in other contexts.[64] These observations support the concept that NOX3 inhibition strategies may be most effective as part of combination approaches targeting complementary sources of oxidative stress.

A distinctive advantage of SRS for VS is that it is a scheduled procedure, most often delivered in a single session, making the timing of the cochlear insult predictable. This stands in contrast to noise trauma or cisplatin ototoxicity, where exposure is either unpredictable or repeated, and thus far more difficult to pre-empt. In this context, a locally delivered intervention could be envisioned prior to SRS. Such delivery might rely on approaches already in clinical development, such as those designed for otoferlin gene therapy via the round window membrane, or on emerging technologies, including double-lumen microneedles that enable simultaneous aspiration and injection to improve intracochlear distribution.[65,66] Importantly, the siRNA construct used in this study was designed and experimentally validated in prior work to target a sequence conserved across the mouse, guinea pig, and human NOX3 orthologs, and was shown to potently inhibit NOX3 expression and activity, underscoring its translational potential for clinical application.[18] Together, these features make siRNA-mediated NOX3 inhibition a particularly promising candidate for translation into an otoprotective therapy administered in conjunction with SRS.

By combining genetic deletion and siRNA-mediated knockdown, this study provides converging evidence that NOX3 inhibition mitigates radiation-induced cochlear injury. Nevertheless, several considerations should be noted when interpreting these findings. All experiments were conducted in the C57BL/6J strain, which carries the cadherin 23 allele linked to age-related hearing loss.[67] Although we used young mice and the contralateral ear as an internal control, this genetic background may influence susceptibility at later time points and should be considered when extrapolating to other strains or species. In addition, while we have previously shown that this SRS protocol induces progressive hearing loss up to 16 weeks,[17] the present study was limited to a 4-week follow-up to avoid confounding by age-related decline. Longer-term studies will be required to determine whether NOX3 inhibition provides durable protection. Although the degree of *Nox3* suppression was not directly re-evaluated in the present cohort, the study builds on a previously validated platform, as both siRNA-mediated knockdown of *Nox3* and the intracochlear delivery approach were extensively characterized in prior work using identical constructs, the same mouse strain, and comparable experimental

Figure 5. Oxidative stress and macrophage response in cochlear cryosections at 4 weeks post-irradiation in siRNA-treated mice

(A and B) Percentage of 4-hydroxynonenal (4-HNE)-positive area in the organ of Corti across apical, middle, and basal turns in scrambled control siRNA (siScr; A; $n = 4$) and siRNA targeting *Nox3* (siNox3; B; $n = 4$) groups, relative to the contralateral cochlea. Increased 4-HNE immunoreactivity was observed in siScr-treated mice, particularly in the middle and basal turns, whereas no increase was observed in siNox3-treated animals. (C) Representative 4-HNE immunofluorescence images of the organ of Corti from basal and middle cochlear turns in siScr- and siNox3-treated mice. Scale bars, 20 μm. (D and E) Percentage of 4-HNE-positive area in the stria vascularis in siScr (D; $n = 4$) and siNox3 (E; $n = 4$) groups, showing increased 4-HNE immunoreactivity in the basal turn of siScr-treated mice and no significant interaural difference in the siNox3-treated mice. (F) Representative 4-HNE immunofluorescence images of the basal turn stria vascularis. Dashed lines delineate the region of interest. Scale bars, 50 μm. (G and H) Iba1-positive cell density (cells/mm^2) in spiral ganglion neurons across cochlear turns in siScr (G; $n = 4$) and siNox3 (H; $n = 4$) groups. Increased macrophage infiltration was observed in all turns of siScr-treated mice and was absent in siNox3-treated animals. (I) Representative Iba1 immunofluorescence images of the basal turn spiral ganglion neurons. Scale bars, 50 μm. (J and K) Iba1-positive cell density in the spiral ligament in siScr (J; $n = 4$) and siNox3 (K; $n = 4$) groups. Increased macrophage infiltration was observed across cochlear turns in siScr-treated mice, whereas siNox3 treatment abolished the increase in the basal turn and attenuated it in the apical and middle turns. (L) Representative Iba1 immunofluorescence images of the basal spiral ligament in siScr- and siNox3-treated groups. Dashed lines delineate the region of interest. Lower panels show magnified views of the boxed areas corresponding to the inferior portion of the spiral ligament. Scale bars, 100 μm. Data are presented as means with error bars representing SEM. Asterisks indicate statistical significance (*$p < 0.05$, **$p < 0.01$, and ***$p < 0.001$). Statistical analysis was performed using two-way repeated measures ANOVA with Bonferroni correction for post hoc pairwise comparisons.

conditions.[18] In the present study, siRNA treatment was timed to coincide with the previously established peak of *Nox3* knockdown, and endpoint analyses at week 4 were prioritized to assess functional protection, cochlear morphology, oxidative stress, and macrophage infiltration. Future studies including early post-irradiation timepoints and clinically relevant delivery approaches could further define radiation-induced *Nox3* expression dynamics, the duration of siRNA-mediated suppression, biodistribution, systemic exposure, and potential off-target or delivery-related safety. Auditory function was assessed using ABR; complementary techniques, such as distortion product otoacoustic emissions (DPOAEs), could provide additional resolution of OHC-specific function, although the combined functional and histological analyses performed here support the main conclusions. The present model was intentionally designed to isolate SRS-induced cochlear injury while avoiding additional variables introduced by VS growth. Although orthotopic implantation models of schwannoma cells into the cerebellopontine angle are feasible,[68] they inherently combine tumor- and radiation-related effects, which can complicate mechanistic interpretation and SRS dose standardization. Future studies incorporating tumor-bearing models will help extend these findings in a more translational setting and determine whether NOX3 inhibition preserves cochlear function without altering tumor response to SRS. Ribbon synapse quantification was based on CtBP2 labeling of presynaptic ribbons, a widely accepted approach for assessing cochlear synaptic structures. This analysis therefore reflects presynaptic ribbon abundance rather than complete synaptic pairing, as post-synaptic markers such as GluR2 were not included. Future dual-labeling studies will further refine the assessment of synaptic integrity. Finally, vestibular function was not assessed; given that SRS can affect balance in patients,[4,69] future work should evaluate vestibular end organs and reflexes and determine whether NOX3 inhibition confers parallel protection in the vestibular system.

In conclusion, our findings support NOX3 inhibition as a targeted and mechanistically grounded strategy to prevent radiation-induced cochlear injury. A successful otoprotective approach in this context should meet three essential criteria: it must act upstream at the source of ROS generation, avoid interfering with the therapeutic effects of SRS on the tumor, and be delivered at the appropriate time, which is feasible given the predictable timing of radiosurgery. This study provides a foundation for developing such interventions and advancing them toward clinical translation.

MATERIALS AND METHODS

Ethical approval

All animal procedures were carried out in accordance with Swiss federal and cantonal legislation on animal welfare and were approved by the Federal Food Safety and Veterinary Office (authorization number 36005) and the Cantonal Veterinary Office of Geneva (authorization number GE316).

Experimental animals and study design

C57BL/6J mice (n = 57), 6 weeks old at baseline, including both males and females, were used in this study. *Nox3* mutant mice (C57BL/6J-$Nox3^{het-4J}$/J; strain #:005014) were obtained from The Jackson Laboratory. These mice carry an ENU-induced splice-site mutation in the *Nox3* gene, leading to aberrant splicing and loss of functional NOX3 expression.[70] Homozygous mutants exhibit a characteristic vestibular phenotype due to the absence of otoconia, while auditory function and cochlear structure are preserved.[71] Colonies were maintained at the animal facility of the University of Geneva through heterozygous × heterozygous breeding, generating wild-type, *Nox3* heterozygous, and *Nox3*-deficient littermates in expected Mendelian ratios. Genotyping was performed for all animals prior to inclusion using PCR-based analysis of DNA obtained from pinna punch biopsies. Mice were group-housed in same-sex groups under standard laboratory conditions (12:12 h light/dark cycle, controlled temperature, and humidity) with unrestricted access to food and water. All animals underwent at least one week of acclimatization before baseline assessments.

Animals were studied in two consecutive experimental arms: (1) the *Nox3* genotype study, which included wild-type ($Nox3^{+/+}$; n = 8), heterozygous ($Nox3^{+/-}$; n = 8), and *Nox3*-deficient ($Nox3^{-/-}$; n = 8) mice (Figure 6A) and (2) the siRNA study, where wild-type mice were randomly assigned to receive either a scrambled siRNA (siScr; n = 16) or an siRNA targeting *Nox3* mRNA (siNox3; n = 17) via the PSCC approach (Figure 6B). In the *Nox3* genotype study, 9 males and 15 females were included, while in the siRNA study, 17 males and 16 females were included.

All procedures associated with potential discomfort, including PSCC injection for siRNA delivery, auditory testing, and SRS, were performed under general anesthesia induced by intraperitoneal injection of ketamine (100 mg/kg; Ketasol, Graeub) and xylazine (10 mg/kg; Rompun, Elanco), diluted in sterile saline to a total volume of 10 μL/g body weight. Anesthesia depth was assessed regularly via pedal withdrawal reflex, and a supplemental intramuscular dose of ketamine (100 mg/kg; 5 μL/g body weight) was administered when needed to maintain adequate anesthesia. During anesthesia, corneal hydration was maintained with ophthalmic ointment. For PSCC injection, local infiltration with lidocaine (0.1 mg/kg; Lidokain, Streuli Pharma AG) was applied at the surgical site, and systemic analgesia was provided via subcutaneous injection of buprenorphine (0.1 mg/kg; Bupaq, Streuli Pharma AG) 30 min before the procedure and again 6 h later.

At the end of the study (week 4), mice were euthanized under deep anesthesia by decapitation without prior fixative administration, in compliance with approved protocols.

siRNA inner ear delivery

As previously reported,[18] we screened 10 distinct siRNA candidates in three cell lines engineered to express the NOX3 complex, including systems expressing human NOX3. We identified the most potent sequence, which exhibited strong inhibitory activity (IC_{50} ≈ 0.3 nM) and a conserved target site across mouse, guinea pig, and human NOX3 orthologs. In addition, functional validation

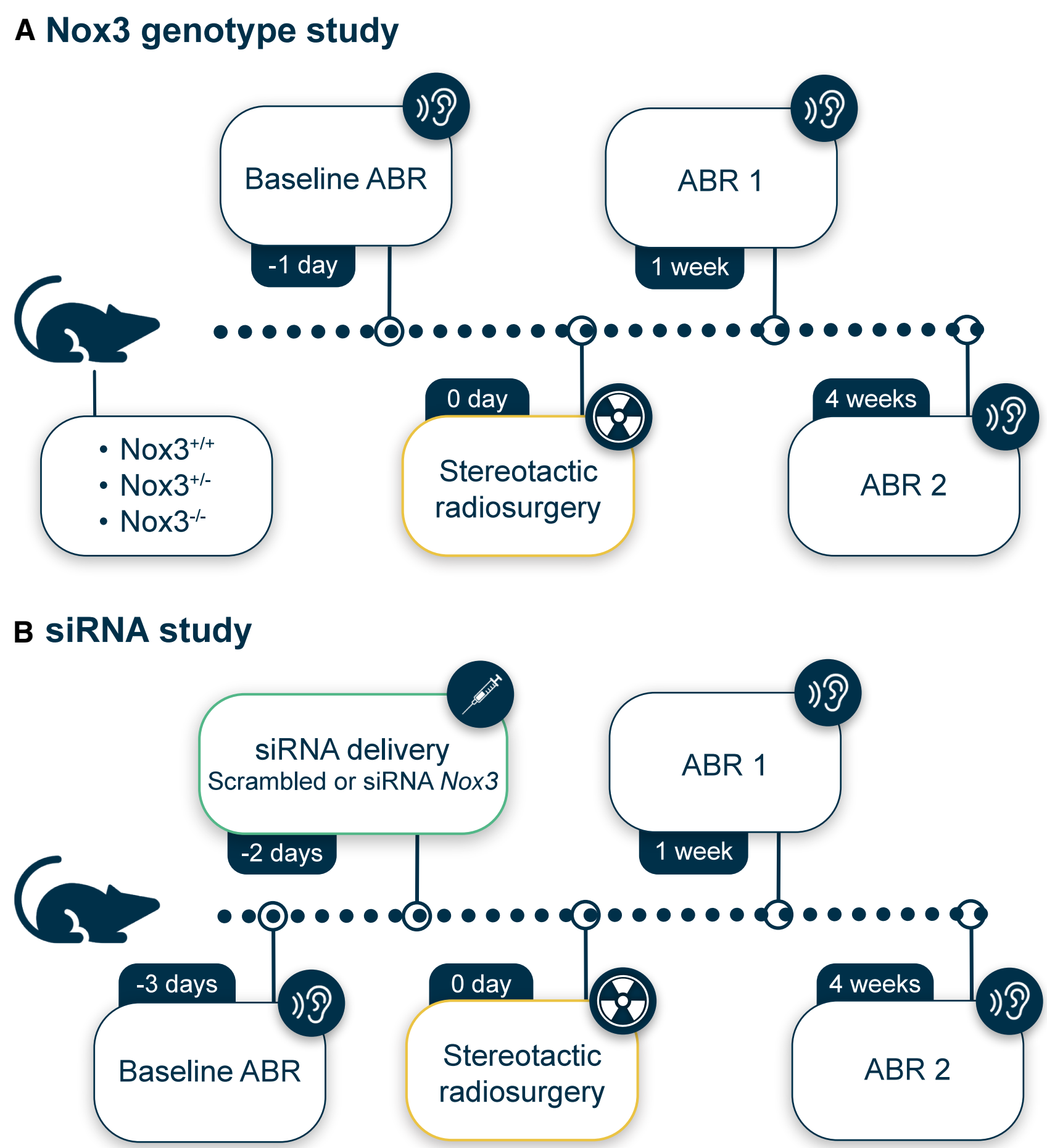

Figure 6. Experimental timelines of the Nox3 genotype and siRNA studies

(A) *Nox3* genotype study. Wild-type (*Nox3*$^{+/+}$; *n* = 8), heterozygous (*Nox3*$^{+/-}$; *n* = 8), and *Nox3*-deficient (*Nox3*$^{-/-}$; *n* = 8) mice underwent auditory brainstem response (ABR) testing in both ears one day before stereotactic radiosurgery (SRS), and at 1 and 4 weeks post-irradiation. Mice were then sacrificed for histological analysis. (B) siRNA study. Baseline ABR measurements were performed in both ears of wild-type mice three days before SRS. Two days prior to SRS, mice were randomly assigned to receive either scrambled siRNA (*n* = 16) or siRNA targeting *Nox3* (*n* = 17) via posterior semicircular canal injection. Hearing was assessed by ABR at 1 and 4 weeks after SRS, followed by sacrifice and histological analysis.

confirmed reduced NOX3-dependent superoxide production and preserved cell viability in NOX3-expressing cell lines following siNox3 treatment. This construct was subsequently tested *in vivo*, where it induced a significant reduction of *Nox3* expression (≈70% knockdown), 48 h after PSCC injection, without affecting hearing function.[18] In that study, intracochlear biodistribution was directly assessed using fluorescently labeled siRNA (siGLO), demonstrating efficient delivery to key cochlear structures, including the organ of Corti and SGNs, following PSCC injection. These validation experiments, including molecular knockdown, specificity assessment, functional assays, and intracochlear biodistribution analysis, were performed in our previous work,[18] and the same construct, delivery protocol, and experimental conditions were used in the present study.

For the present study, this validated siNox3 construct (Decibel Therapeutics) or a non-targeting siScr control (Thermo Fisher Scientific, AM4611) was pre-complexed with a cationic lipid formulation to facilitate cellular uptake. siRNAs (40 μM) were diluted 1:1 in Opti-MEM (Thermo Fisher Scientific, 31985062) and incubated with Lipofectamine RNAiMAX (Thermo Fisher Scientific, 13778100) following the manufacturer's protocol. The final solution contained 10 μM siRNA and was used for direct intracochlear injection.

siRNA delivery into the inner ear was performed via PSCC as previously described by Suzuki et al.[72] and adapted in our laboratory[18] 48 h before SRS (Figure S6). Mice were anesthetized with ketamine/xylazine and placed in a lateral position under a surgical microscope. After retroauricular incision and muscle dissection, the PSCC was exposed and carefully fenestrated with a 25G needle. The canal was then catheterized using a polyimide surgical microtube (MicroLumen, 039-I) connected to a 25 μL syringe (Hamilton, 80465). The bone-tube interface was sealed with muscle tissue and cyanoacrylate to prevent reagent leakage. A volume of 1 μL of siNox3 or siScr solution was slowly injected over 2 min by steady manual compression of the syringe plunger. To promote intracochlear diffusion, the animal was kept in the lateral position for an additional 10 min before carefully removing the injection system. The canal opening was then sealed with tissue and cyanoacrylate. The incision site was sutured, disinfected with iodine solution, and the animals received local lidocaine as previously described.

Stereotactic radiosurgery protocol

Precise 32 Gy unilateral cochlear irradiation was performed using the Leksell Gamma Knife ICON system (Elekta AB, Stockholm, Sweden), a clinical-grade SRS platform, following our previously validated protocol.[17] This setup reliably induces high-frequency hearing loss by 4 weeks post-SRS, with corresponding histological damage in the irradiated cochlea, while sparing contralateral auditory structures.

Mice were anesthetized with ketamine/xylazine and positioned prone on a custom-designed thermoplastic stereotactic frame. For each experimental animal, an initial cone-beam computed

tomography (CBCT) scan was acquired and co-registered with a high-resolution CT-based anatomical atlas and a mouse brain magnetic resonance imaging (MRI) atlas to accurately identify inner ear structures and surrounding anatomy.[73,74] Bilateral segmentation of the cochleae was performed using the co-registered images to generate individualized three-dimensional volumes of interest for each animal. Treatment planning was performed with the Leksell GammaPlan software (Elekta AB, Stockholm, Sweden), positioning a single isocenter lateral to the right cochlea. The isocenter was positioned so that the 80% isodose line, corresponding to the 32 Gy dose prescription, was aligned to cover the medial edge of the right cochlea while minimizing exposure to adjacent structures.

This protocol ensures spatially precise and reproducible irradiation of the targeted cochlea while minimizing damage to the central auditory pathways and sparing the contralateral cochlea, which serves as an internal control.[17]

Auditory brainstem response testing

Baseline ABR measurements were performed one day before irradiation (*Nox3* genotype study) and one day before PSCC injection (siRNA study). Follow-up measurements were performed at 1 and 4 weeks post-SRS (Figure 6). Recordings took place in a sound-attenuated chamber (IAC Acoustics, IL, USA) under general anesthesia, as described above. Mice were placed on an isothermal pad to maintain body temperature throughout testing.

Acoustic stimuli were delivered independently to each ear using custom-fitted adapters, with the contralateral ear serving as an internal control. Subdermal needle electrodes were placed at the vertex (active), the ipsilateral mastoid (reference), and the back of the experimental animal (ground). Stimulus generation and signal acquisition were controlled using a multifunction I/O card (National Instruments), and sound output was calibrated online before each measurement using a probe microphone system (Brüel & Kjær 4191). Signals were amplified (80 dB) and band-pass filtered (0.2–3.0 kHz) before averaging.

Each session included click stimuli (100 μs broadband pulses) and pure tone bursts (2–32 kHz; two steps per octave; 3 ms duration), each presented and averaged from 128 repetitions. Click stimuli were delivered in 3 dB sound pressure level (SPL) steps and tone bursts in 5 dB SPL steps, from 0 to 90 dB SPL. ABR threshold was defined as the lowest sound level eliciting a consistent, reproducible waveform.

Wave amplitudes (I, III, and IV) were calculated as the voltage difference between the positive peak of each wave and its subsequent negative trough, in response to 80 dB SPL click and tone burst stimuli at 8, 16, and 32 kHz. Following testing, mice were monitored on a heating pad until full recovery.

Cochlear dissection and preparation

At week 4, following the final ABR assessment, mice were euthanized, and the temporal bones were promptly collected to prevent postmortem degradation. Under a stereomicroscope, the auditory bulla was dissected to expose the cochlea, which was immediately transferred to cold PBS. All cochleae were visually inspected to confirm the absence of middle ear effusion and mechanical damage.

Cochleae were fixed overnight at 4°C in 4% paraformaldehyde, then decalcified for 48 h at room temperature using a commercial decalcifying solution (USEDECALC, Art. No. 40-3310-00), with a fresh solution applied after 24 h. After decalcification, cochleae from each experimental animal were randomly assigned to one of three processing workflows: (1) whole-mount dissection of the sensory epithelium for immunofluorescence studies, (2) paraffin embedding for mid-modiolar sectioning, or (3) cryoprotection and embedding in optimal cutting temperature (OCT) compound for cryosection-based immunofluorescence.

Cochlear histology

Whole-mount immunohistochemistry and confocal microscopy

Decalcified cochleae designated for whole-mount analysis were dissected into basal, middle, and apical turns as previously described.[75] Sensory epithelium segments were adhered to 12 mm coverslips using Cell-Tak (Corning, Cat# CLS354240) and stored in PBS at 4°C prior to staining.[76]

Samples were permeabilized in 2% Triton X-100 in PBS for 30 min at room temperature, then incubated in 2% BSA and 0.01% Triton X-100 in PBS for 30 min. The primary antibodies, rabbit anti-Myosin VIIa (1:200, Proteus Biosciences, Cat# 25–6790) and mouse anti-CtBP2 (1:200, BD Biosciences, Cat# 612044), were applied overnight at 4°C. After three PBS rinses, the secondary antibodies donkey anti-rabbit Alexa Fluor 488 (1:500; Thermo Fisher Scientific, Cat# A21206) and donkey anti-mouse Alexa Fluor 555 (1:500; Thermo Fisher Scientific, Cat# A31570) were incubated for 2 h at room temperature. Following three PBS rinses, tissues were mounted on glass slides using DAPI-containing mounting medium (Fluoroshield Sigma, Cat# F6057).

Image acquisition was performed using a Zeiss LSM800 confocal microscope. For hair cell quantification, Z-stacks were captured with a Plan-Apochromat 20×/0.8 objective (pixel size: 0.624 μm; step size: 3 μm) and stitched using the Tiles function to reconstruct entire cochlear segments. Higher-resolution images for figures were taken with the same objective at 0.156 μm pixel size and 0.49 μm step size. Ribbon synapses were imaged using a Plan-Apochromat 63×/1.4 oil immersion objective (pixel size: 0.099 μm; step size: 0.5 μm) over 10–15 μm Z-stacks.

Mid-modiolar hematoxylin and eosin staining

Cochleae designated for mid-modiolar analysis were dehydrated through a graded ethanol series and embedded in paraffin. Serial 5 μm mid-modiolar sections were collected onto glass slides. Hematoxylin and eosin (H&E) staining was performed using Harris' hematoxylin (5 min), followed by differentiation in acid alcohol, and counterstaining with eosin (2–3 s). Sections were dehydrated, cleared in xylene, and mounted with Eukitt medium.

Images were acquired with a Zeiss Axio Scan.Z1 slide scanner using a Plan-Apochromat 20×/0.8 objective (pixel size: 0.22 μm).

Immunofluorescence on cochlear cryosections

Decalcified cochleae designated for cryosection analysis were cryoprotected in sucrose solution, embedded in OCT compound, and sectioned at 6 μm thickness using a cryostat. Serial sections were collected on glass slides and stored at −20°C until staining.

For immunofluorescence, sections were brought to room temperature, postfixed in 2% paraformaldehyde for 5 min, washed three times with PBS, and permeabilized with 0.5% Triton X-100 in PBS for 20 min, and washed three times again with PBS. After blocking with 1% BSA in PBS for 30 min, sections were incubated overnight at 4°C with primary antibodies against β3-tubulin (TUJ1; mouse, 1:1000, BioLegend, Cat# 801202; or rabbit, 1:2000, BioLegend, Cat# 802001), 4-hydroxynonenal (4-HNE; 1:200, Abcam, Cat# ab48506), and Iba1 (1:500, Wako, Cat# 019–19741). The β3-tubulin antibody species was selected to ensure compatibility with the co-stained primary antibody. After three PBS washes, sections were incubated for 1 h at room temperature with the appropriate secondary antibodies: donkey anti-rabbit Alexa Fluor 488 (1:1000; Thermo Fisher Scientific, Cat# A21206), donkey anti-mouse Alexa Fluor Plus 488 (1:1000; Thermo Fisher Scientific, Cat# A32766), donkey anti-mouse Alexa Fluor 555 (1:1000; Thermo Fisher Scientific, Cat# A31570), and donkey anti-rabbit Alexa Fluor 555 (1:1000; Thermo Fisher Scientific, Cat# A31572). Sections were then rinsed three times with PBS, counterstained with DAPI-containing mounting medium (Fluoroshield, Sigma), and coverslipped.

Image acquisition was performed using a Zeiss LSM800 confocal microscope (pixel size: 0.31 μm; step size: 0.5 μm for 4-HNE and 1 μm for Iba1). Laser power, gain, and offset were kept constant across all samples. For each cochlea and staining condition, two non-consecutive mid-modiolar sections were imaged, and Z-stacks were acquired for each region of interest. Average intensity projections were used for 4-HNE analysis and maximum intensity projections for Iba1 analysis.

Quantitative analysis of cochlear morphology

Maximum-intensity projections of stitched cytocochleograms were generated from Z-stacks. Apical, middle, and basal turns were assembled into full cochlear views. Using Fiji (ImageJ) and the Measure_Line plugin (Eaton-Peabody Laboratories, Mass Eye and Ear), a line was traced along the lateral margin of the IHC row to measure the total epithelium length and assign frequency-specific regions based on cochlear tonotopy.[77] IHCs and OHCs were manually counted within 300 μm segments centered on mapped frequencies.

CtBP2-positive puncta were counted manually in maximum projections of 10–15 μm confocal Z-stacks. Counts were performed in 10–15 adjacent IHCs at frequency-specific cochlear positions based on the previously generated mask, and results were expressed as mean ribbons per IHC for each frequency region.

From each cochlea, four non-consecutive mid-modiolar H&E-stained sections were analyzed. Rosenthal's canal was manually outlined in QuPath and divided by cochlear region (apex, middle, base). SGN nuclei were quantified using the Cellpose plugin and a custom analysis script. All results were validated manually and normalized to canal area (SGN/mm^2). Mean values per turn were used for group comparisons.

Stria thickness was measured in four non-consecutive mid-modiolar sections per cochlea. A linear measurement was taken at each turn (apex, middle, and base) from the outer edge of the stria to the basal cell-spiral ligament junction, midway between Reissner's membrane attachment and the spiral prominence.[78] Mean values were calculated per turn for statistical analysis.

Quantification of 4-HNE immunoreactivity was performed in Fiji (ImageJ) by measuring the percentage of positive area within predefined regions of interest, including SGNs, organ of Corti, and stria vascularis. A fixed threshold was applied to all images based on negative control sections processed without the primary antibody. Iba1-positive cells were manually counted within predefined regions of interest, including SGNs, spiral ligament, and stria vascularis. Cell density was calculated by normalizing counts to the corresponding ROI area and expressed as cells/mm^2.[79] For both analyses, values from two non-consecutive sections per cochlea were averaged. Mean values per cochlear turn were used for group comparisons.

Statistical analysis

Statistical analyses were performed using GraphPad Prism (version 10.5). Two-way repeated measures ANOVA was used to compare ABR threshold shifts and histological parameters. For within-animal comparisons, the ear (irradiated vs. contralateral) was treated as the repeated measure, and frequency or cochlear turn as the within-subject factor. For between-group comparisons at week 4, the treatment group was the between-subject factor, and frequency was the repeated measure. Interaction terms were included in all models. Bonferroni correction was applied for multiple comparisons. Normality and sphericity were verified. For comparisons of ABR wave amplitudes between groups, the Mann-Whitney test was employed. A two-sided $p < 0.05$ was considered statistically significant.

DATA AND CODE AVAILABILITY

The data supporting the findings of this study are available from the corresponding author upon reasonable request.

ACKNOWLEDGMENTS

We gratefully acknowledge Nicolas Liaudet and Sergei Startchik from the Bioimaging Core Facility of the University of Geneva for their expert support in imaging analysis. We also thank Cristian Cotrutz from the Lausanne Gamma Knife Center for his valuable assistance in developing the stereotactic irradiation protocol, and Nicolas Dupuy for his help during animal experimentation at the same facility. The Swiss Academy of Medical Sciences MD-PhD grant (323630_214546) supported D.D. An Eccellenza fellowship from the Swiss National Science Foundation (194260) supported I.O.J. A Swiss National Science Foundation grant (204546) supported F.R. and P.S.

AUTHOR CONTRIBUTIONS

D.D., conceptualization, methodology, investigation, formal analysis, visualization, writing – original draft, and writing – review and editing; F.R., conceptualization, methodology, investigation, formal analysis, resources, visualization, writing – original draft, and writing – review and editing; S.S., investigation and writing – review and editing; L.O., investigation and writing – review and editing; J.-P.T., conceptualization, methodology, and writing – review and editing; C.T., methodology and writing – review and editing; I.O.J., conceptualization, methodology, and writing – review and editing; M.L., conceptualization, methodology, investigation, formal analysis, resources, writing – review and editing, and supervision; P.S., conceptualization, methodology, formal analysis, resources, writing – review and editing, and supervision.

DECLARATION OF INTERESTS

The authors declare no competing interests.

DECLARATION OF GENERATIVE AI AND AI-ASSISTED TECHNOLOGIES IN THE WRITING PROCESS

During the preparation of this work, the authors used ChatGPT (OpenAI) to improve readability and language. After using this tool/service, the authors reviewed and edited the content as needed and take full responsibility for the content of the published article.

SUPPLEMENTAL INFORMATION

Supplemental information can be found online at https://doi.org/10.1016/j.omtn.2026.103036.

REFERENCES

1. Carlson, M.L., and Link, M.J. (2021). Vestibular Schwannomas. N. Engl. J. Med. *384*, 1335–1348. https://doi.org/10.1056/NEJMra2020394.
2. Marinelli, J.P., Beeler, C.J., Carlson, M.L., Caye-Thomasen, P., Spear, S.A., and Erbele, I.D. (2022). Global Incidence of Sporadic Vestibular Schwannoma: A Systematic Review. Otolaryngol. Head Neck Surg. *167*, 209–214. https://doi.org/10.1177/01945998211042006.
3. Goldbrunner, R., Weller, M., Regis, J., Lund-Johansen, M., Stavrinou, P., Reuss, D., Evans, D.G., Lefranc, F., Sallabanda, K., Falini, A., et al. (2020). EANO guideline on the diagnosis and treatment of vestibular schwannoma. Neuro Oncol. *22*, 31–45. https://doi.org/10.1093/neuonc/noz153.
4. Balossier, A., Tuleasca, C., Delsanti, C., Troude, L., Thomassin, J.M., Roche, P.H., and Régis, J. (2023). Long-Term Hearing Outcome After Radiosurgery for Vestibular Schwannoma: A Systematic Review and Meta-Analysis. Neurosurgery *92*, 1130–1141. https://doi.org/10.1227/neu.0000000000002354.
5. Coughlin, A.R., Willman, T.J., and Gubbels, S.P. (2018). Systematic Review of Hearing Preservation After Radiotherapy for Vestibular Schwannoma. Otol. Neurotol. *39*, 273–283. https://doi.org/10.1097/mao.0000000000001672.
6. Peris-Celda, M., Graffeo, C.S., Perry, A., Kleinstern, G., Kerezoudis, P., Driscoll, C.L.W., Carlson, M.L., and Link, M.J. (2020). Beyond the ABCs: Hearing Loss and Quality of Life in Vestibular Schwannoma. Mayo Clin. Proc. *95*, 2420–2428. https://doi.org/10.1016/j.mayocp.2020.03.033.
7. Hoistad, D.L., Ondrey, F.G., Mutlu, C., Schachern, P.A., Paparella, M.M., and Adams, G.L. (1998). Histopathology of human temporal bone after cis-platinum, radiation, or both. Otolaryngol. Head Neck Surg. *118*, 825–832. https://doi.org/10.1016/s0194-5998(98)70276-1.
8. Low, W.K., Burgess, R., Fong, K.W., and Wang, D.Y. (2005). Effect of radiotherapy on retro-cochlear auditory pathways. Laryngoscope *115*, 1823–1826. https://doi.org/10.1097/01.mlg.0000175061.59315.58.
9. Gao, Y., Wu, F., He, W., Cai, Z., Pang, J., and Zheng, Y. (2024). ROS-related disruptions to cochlear hair cell and stria vascularis consequently leading to radiation-induced sensorineural hearing loss. Antioxid. Redox Signal. *40*, 470–491. https://doi.org/10.1089/ars.2022.0161.
10. Mujica-Mota, M.A., Lehnert, S., Devic, S., Gasbarrino, K., and Daniel, S.J. (2014). Mechanisms of radiation-induced sensorineural hearing loss and radioprotection. Hear. Res. *312*, 60–68. https://doi.org/10.1016/j.heares.2014.03.003.
11. Shi, W., Hou, X., Bao, X., Hou, W., Jiang, X., Ma, L., Jiang, X., and Dong, L. (2021). Mechanism and Protection of Radiotherapy Induced Sensorineural Hearing Loss for Head and Neck Cancer. Biomed Res. Int. *2021*, 3548706. https://doi.org/10.1155/2021/3548706.
12. Cipriano, A., Viviano, M., Feoli, A., Milite, C., Sarno, G., Castellano, S., and Sbardella, G. (2023). NADPH Oxidases: From Molecular Mechanisms to Current Inhibitors. J. Med. Chem. *66*, 11632–11655. https://doi.org/10.1021/acs.jmedchem.3c00770.
13. Mukherjea, D., Jajoo, S., Kaur, T., Sheehan, K.E., Ramkumar, V., and Rybak, L.P. (2010). Transtympanic administration of short interfering (si)RNA for the NOX3 isoform of NADPH oxidase protects against cisplatin-induced hearing loss in the rat. Antioxid. Redox Signal. *13*, 589–598. https://doi.org/10.1089/ars.2010.3110.
14. Rousset, F., Nacher-Soler, G., Coelho, M., Ilmjarv, S., Kokje, V.B.C., Marteyn, A., Cambet, Y., Perny, M., Roccio, M., Jaquet, V., et al. (2020). Redox activation of excitatory pathways in auditory neurons as mechanism of age-related hearing loss. Redox Biol. *30*, 101434. https://doi.org/10.1016/j.redox.2020.101434.
15. Rousset, F., Nacher-Soler, G., Kokje, V.B.C., Sgroi, S., Coelho, M., Krause, K.H., and Senn, P. (2022). NADPH Oxidase 3 Deficiency Protects From Noise-Induced Sensorineural Hearing Loss. Front. Cell Dev. Biol. *10*, 832314. https://doi.org/10.3389/fcell.2022.832314.
16. Pyun, J.H., Kang, S.U., Hwang, H.S., Oh, Y.T., Kang, S.H., Lim, Y.A., Choo, O.S., and Kim, C.H. (2011). Epicatechin inhibits radiation-induced auditory cell death by suppression of reactive oxygen species generation. Neuroscience *199*, 410–420. https://doi.org/10.1016/j.neuroscience.2011.09.012.
17. Daskalou, D., Rousset, F., Sgroi, S., Oberhauser, L., Thiran, J.P., Tuleasca, C., Jelescu, I.O., Levivier, M., and Senn, P. (2026). Introducing a translationally relevant mouse model of radiosurgery-induced unilateral hearing loss. Front. Neurosci. *20*, 1748309. https://doi.org/10.3389/fnins.2026.1748309.
18. Nacher-Soler, G., Marteyn, A., Barenzung, N., Sgroi, S., Krause, K.H., Senn, P., and Rousset, F. (2022). Development and in vivo validation of small interfering RNAs targeting NOX3 to prevent sensorineural hearing loss. Front. Neurol. *13*, 993017. https://doi.org/10.3389/fneur.2022.993017.
19. Dinh, C.T., Chen, S., Dinh, J., Goncalves, S., Bas, E., Padgett, K., Johnson, P., Elsayyad, N., Telischi, F., and Van De Water, T. (2017). Effects of Intratympanic Dexamethasone on High-Dose Radiation Ototoxicity In Vivo. Otol. Neurotol. *38*, 180–186. https://doi.org/10.1097/MAO.0000000000001289.
20. Liu, J., Zhu, L., Bao, Y., Du, Z., Shi, L., Hong, X., Zou, Z., and Peng, G. (2024). Injectable dexamethasone-loaded peptide hydrogel for therapy of radiation-induced ototoxicity by regulating the mTOR signaling pathway. J. Control. Release *365*, 729–743. https://doi.org/10.1016/j.jconrel.2023.12.004.
21. Liu, Z., Luo, Y., Guo, R., Yang, B., Shi, L., Sun, J., Guo, W., Gong, S., Jiang, X., and Liu, K. (2022). Head and neck radiotherapy causes significant disruptions of cochlear ribbon synapses and consequent sensorineural hearing loss. Radiother. Oncol. *173*, 207–214. https://doi.org/10.1016/j.radonc.2022.05.023.
22. Gasser Rutledge, K.L., Prasad, K.G., Emery, K.R., Mikulec, A.A., Varvares, M., and Gratton, M.A. (2015). Short-term Peripheral Auditory Effects of Cranial Irradiation: A Mouse Model. Ann. Otol. Rhinol. Laryngol. *124*, 903–910. https://doi.org/10.1177/0003489415591205.
23. Karaer, I., Simsek, G., Gul, M., Bahar, L., Gürocak, S., Parlakpinar, H., and Nuransoy, A. (2015). Melatonin protects inner ear against radiation damage in rats. Laryngoscope *125*, E345–E349. https://doi.org/10.1002/lary.25376.
24. Baker, K., and Staecker, H. (2012). Low Dose Oxidative Stress Induces Mitochondrial Damage in Hair Cells. Anat. Rec. *295*, 1868–1876. https://doi.org/10.1002/ar.22594.
25. Eriksson, D., and Stigbrand, T. (2010). Radiation-induced cell death mechanisms. Tumour Biol. *31*, 363–372. https://doi.org/10.1007/s13277-010-0042-8.
26. Havaki, S., Kotsinas, A., Chronopoulos, E., Kletsas, D., Georgakilas, A., and Gorgoulis, V.G. (2015). The role of oxidative DNA damage in radiation induced bystander effect. Cancer Lett. *356*, 43–51. https://doi.org/10.1016/j.canlet.2014.01.023.
27. Kryston, T.B., Georgiev, A.B., Pissis, P., and Georgakilas, A.G. (2011). Role of oxidative stress and DNA damage in human carcinogenesis. Mutat. Res. *711*, 193–201. https://doi.org/10.1016/j.mrfmmm.2010.12.016.

28. Tabuchi, K., Nishimura, B., Nakamagoe, M., Hayashi, K., Nakayama, M., and Hara, A. (2011). Ototoxicity: mechanisms of cochlear impairment and its prevention. Curr. Med. Chem. *18*, 4866–4871. https://doi.org/10.2174/092986711797535254.

29. Sobotta, M.C., Liou, W., Stöcker, S., Talwar, D., Oehler, M., Ruppert, T., Scharf, A.N.D., and Dick, T.P. (2015). Peroxiredoxin-2 and STAT3 form a redox relay for H2O2 signaling. Nat. Chem. Biol. *11*, 64–70. https://doi.org/10.1038/nchembio.1695.

30. Chen, Y., and Shen, Y.Q. (2025). Role of reactive oxygen species in regulating epigenetic modifications. Cell. Signal. *125*, 111502. https://doi.org/10.1016/j.cellsig.2024.111502.

31. Hong, Y., Boiti, A., Vallone, D., and Foulkes, N.S. (2024). Reactive Oxygen Species Signaling and Oxidative Stress: Transcriptional Regulation and Evolution. Antioxidants *13*, 312. https://doi.org/10.3390/antiox13030312.

32. Lu, C., Chan, S.L., Fu, W., and Mattson, M.P. (2002). The lipid peroxidation product 4-hydroxynonenal facilitates opening of voltage-dependent Ca2+ channels in neurons by increasing protein tyrosine phosphorylation. J. Biol. Chem. *277*, 24368–24375. https://doi.org/10.1074/jbc.M201924200.

33. Shi, M., Wang, Y., Yang, H., Lai, C., Yu, J., and Sun, Y. (2024). High doses of radiation cause cochlear immunological stress and sensorineural hearing loss. Heliyon *10*, e37223. https://doi.org/10.1016/j.heliyon.2024.e37223.

34. Chen, T., Luo, Y., Li, Q., Yang, C., Yuan, Y., Peng, J., Ban, M., Liang, Y., and Zhang, W. (2021). Melatonin reduces radiation damage in inner ear. J. Radiat. Res. *62*, 217–225. https://doi.org/10.1093/jrr/rraa137.

35. Altas, E., Ertekin, M.V., Gundogdu, C., and Demirci, E. (2006). L-carnitine reduces cochlear damage induced by gamma irradiation in Guinea pigs. Ann. Clin. Lab. Sci. 36, 312–318.

36. Lessa, R.M., Oliveira, J.A.A.d., Rossato, M., and Ghilardi Netto, T. (2009). Analysis of the cytoprotective effect of amifostine on the irradiated inner ear of guinea pigs: an experimental study. Braz. J. Otorhinolaryngol. *75*, 694–700. https://doi.org/10.1016/s1808-8694(15)30520-6.

37. Tong, J., Hu, C., Wu, Y., Liu, Q., and Sun, D. (2023). Radiation-induced NF-kappaB activation is involved in cochlear damage in mice via promotion of a local inflammatory response. J. Radiat. Res. *64*, 63–72. https://doi.org/10.1093/jrr/rrac068.

38. Mujica-Mota, M.A., Salehi, P., Devic, S., and Daniel, S.J. (2014). Safety and otoprotection of metformin in radiation-induced sensorineural hearing loss in the guinea pig. Otolaryngol. Head Neck Surg. *150*, 859–865. https://doi.org/10.1177/0194599814521013.

39. Sahin, M., Kaya, A., Aytekin, A., Akay, E., and Ozcan, I. (2023). Tetramethylpyrazine Attenuates Radiation-Induced Ototoxicity in a Rat Model. Audiol. Neurootol. *28*, 427–435. https://doi.org/10.1159/000530685.

40. Low, W.K., Tan, M.G.K., Sun, L., Chua, A.W.C., Goh, L.K., and Wang, D.Y. (2006). Dose-dependant radiation-induced apoptosis in a cochlear cell-line. Apoptosis *11*, 2127–2136. https://doi.org/10.1007/s10495-006-0285-4.

41. Wang, Y., Liu, Q., Zhao, W., Zhou, X., Miao, G., Sun, C., and Zhang, H. (2017). NADPH Oxidase Activation Contributes to Heavy Ion Irradiation-Induced Cell Death. Dose Response *15*, 1559325817699697. https://doi.org/10.1177/1559325817699697.

42. Zheng, Z., Su, J., Bao, X., Wang, H., Bian, C., Zhao, Q., and Jiang, X. (2023). Mechanisms and applications of radiation-induced oxidative stress in regulating cancer immunotherapy. Front. Immunol. *14*, 1247268. https://doi.org/10.3389/fimmu.2023.1247268.

43. Liu, R., Bian, Y., Liu, L., Liu, L., Liu, X., and Ma, S. (2022). Molecular pathways associated with oxidative stress and their potential applications in radiotherapy. Int. J. Mol. Med. *49*, 65. https://doi.org/10.3892/ijmm.2022.5121.

44. Limbrunner, J., Doerfler, J., Pietschmann, K., Buentzel, J., Scharpenberg, M., and Huebner, J. (2025). The influence of antioxidant supplementation on adverse effects and tumor interaction during radiotherapy: a systematic review. Clin. Exp. Med. *25*, 258. https://doi.org/10.1007/s10238-025-01804-x.

45. Pitter, K.L., Tamagno, I., Alikhanyan, K., Hosni-Ahmed, A., Pattwell, S.S., Donnola, S., Dai, C., Ozawa, T., Chang, M., Chan, T.A., et al. (2016). Corticosteroids compromise survival in glioblastoma. Brain *139*, 1458–1471. https://doi.org/10.1093/brain/aww046.

46. Robinett, Z.N., Bathla, G., Wu, A., Clark, J.J., Sibenaller, Z.A., Wilson, T., Kirby, P., Allen, B.G., and Hansen, M.R. (2018). Persistent Oxidative Stress in Vestibular Schwannomas After Stereotactic Radiation Therapy. Otol. Neurotol. *39*, 1184–1190. https://doi.org/10.1097/MAO.0000000000001935.

47. Di Stadio, A., Ralli, M., Kaski, D., Koohi, N., Gioacchini, F.M., Kysar, J.W., Lalwani, A.K., Warnecke, A., and Bernitsas, E. (2024). Exploring Inner Ear and Brain Connectivity through Perilymph Sampling for Early Detection of Neurological Diseases: A Provocative Proposal. Brain Sci. *14*, 621. https://doi.org/10.3390/brainsci14060621.

48. Salt, A.N., and Hirose, K. (2018). Communication pathways to and from the inner ear and their contributions to drug delivery. Hear. Res. *362*, 25–37. https://doi.org/10.1016/j.heares.2017.12.010.

49. Bánfi, B., Malgrange, B., Knisz, J., Steger, K., Dubois-Dauphin, M., and Krause, K.H. (2004). NOX3, a superoxide-generating NADPH oxidase of the inner ear. J. Biol. Chem. *279*, 46065–46072. https://doi.org/10.1074/jbc.M403046200.

50. Mohri, H., Ninoyu, Y., Sakaguchi, H., Hirano, S., Saito, N., and Ueyama, T. (2021). Nox3-Derived Superoxide in Cochleae Induces Sensorineural Hearing Loss. J. Neurosci. *41*, 4716–4731. https://doi.org/10.1523/jneurosci.2672-20.2021.

51. Bhandare, N., Jackson, A., Eisbruch, A., Pan, C.C., Flickinger, J.C., Antonelli, P., and Mendenhall, W.M. (2010). Radiation therapy and hearing loss. Int. J. Radiat. Oncol. Biol. Phys. *76*, S50–S57. https://doi.org/10.1016/j.ijrobp.2009.04.096.

52. Prayuenyong, P., Baguley, D.M., Kros, C.J., and Steyger, P.S. (2021). Preferential Cochleotoxicity of Cisplatin. Front. Neurosci. *15*, 695268. https://doi.org/10.3389/fnins.2021.695268.

53. Liu, X.Z., and Yan, D. (2007). Ageing and hearing loss. J. Pathol. *211*, 188–197. https://doi.org/10.1002/path.2102.

54. Yang, Y., Chen, X., Tian, C., Fan, B., An, X., Liu, Z., Li, Q., Mi, W., Lin, Y., and Zha, D. (2024). Gene expression analysis of oxidative stress-related genes in the apical, middle, and basal turns of the cochlea. Gene Expr. Patterns *51*, 119356. https://doi.org/10.1016/j.gep.2024.119356.

55. Sha, S.H., Taylor, R., Forge, A., and Schacht, J. (2001). Differential vulnerability of basal and apical hair cells is based on intrinsic susceptibility to free radicals. Hear. Res. *155*, 1–8. https://doi.org/10.1016/s0378-5955(01)00224-6.

56. Sajid, M.I., Moazzam, M., Kato, S., Cho, K.Y., and Tiwari, R.K. (2020). Overcoming Barriers for siRNA Therapeutics: From Bench to Bedside. Pharmaceuticals *13*, 294. https://doi.org/10.3390/ph13100294.

57. Zhang, J., Chen, B., Gan, C., Sun, H., Zhang, J., and Feng, L. (2023). A Comprehensive Review of Small Interfering RNAs (siRNAs): Mechanism, Therapeutic Targets, and Delivery Strategies for Cancer Therapy. Int. J. Nanomed. *18*, 7605–7635. https://doi.org/10.2147/ijn.S436038.

58. Rousset, F., Salmon, P., Bredl, S., Cherpin, O., Coelho, M., Myburgh, R., Alessandrini, M., Perny, M., Roccio, M., Speck, R.F., et al. (2019). Optimizing Synthetic miRNA Minigene Architecture for Efficient miRNA Hairpin Concatenation and Multi-target Gene Knockdown. Mol. Ther. Nucleic Acids *14*, 351–363. https://doi.org/10.1016/j.omtn.2018.12.004.

59. Kamogashira, T., Fujimoto, C., and Yamasoba, T. (2015). Reactive oxygen species, apoptosis, and mitochondrial dysfunction in hearing loss. Biomed Res. Int. *2015*, 617207. https://doi.org/10.1155/2015/617207.

60. Wong, A.C.Y., and Ryan, A.F. (2015). Mechanisms of sensorineural cell damage, death and survival in the cochlea. Front. Aging Neurosci. *7*, 58. https://doi.org/10.3389/fnagi.2015.00058.

61. McFadden, S.L., Ohlemiller, K.K., Ding, D., Shero, M., and Salvi, R.J. (2001). The Influence of Superoxide Dismutase and Glutathione Peroxidase Deficiencies on Noise-Induced Hearing Loss in Mice. Noise Health 3, 49–64.

62. Guo, C., Sun, L., Chen, X., and Zhang, D. (2013). Oxidative stress, mitochondrial damage and neurodegenerative diseases. Neural Regen. Res. *8*, 2003–2014. https://doi.org/10.3969/j.issn.1673-5374.2013.21.009.

63. Brandes, R.P., Weissmann, N., and Schröder, K. (2014). Nox family NADPH oxidases: Molecular mechanisms of activation. Free Radic. Biol. Med. *76*, 208–226. https://doi.org/10.1016/j.freeradbiomed.2014.07.046.

64. Fujimoto, C., and Yamasoba, T. (2019). Mitochondria-Targeted Antioxidants for Treatment of Hearing Loss: A Systematic Review. Antioxidants *8*, 109. https://doi.org/10.3390/antiox8040109.

65. Lustig, L. (2025). Cochlear gene therapy for otoferlin-related hearing loss. Curr. Opin. Otolaryngol. Head Neck Surg. *33*, 312–317. https://doi.org/10.1097/moo.0000000000001070.

66. Hammer, D.R., Voruz, F., Aksit, A., Breil, E., Rousset, F., Senn, P., Ilmjärv, S., Olson, E.S., Lalwani, A.K., and Kysar, J.W. (2025). Novel dual-lumen microneedle delivers adeno-associated viral vectors in the guinea pig inner ear via the round window membrane. Biomed. Microdevices *27*, 27. https://doi.org/10.1007/s10544-025-00751-4.

67. Yasuda, S.P., Seki, Y., Suzuki, S., Ohshiba, Y., Hou, X., Matsuoka, K., Wada, K., Shitara, H., Miyasaka, Y., and Kikkawa, Y. (2020). c.753A>G genome editing of a Cdh23ahl allele delays age-related hearing loss and degeneration of cochlear hair cells in C57BL/6J mice. Hear. Res. *389*, 107926. https://doi.org/10.1016/j.heares.2020.107926.

68. Chen, J., Landegger, L.D., Sun, Y., Ren, J., Maimon, N., Wu, L., Ng, M.R., Chen, J.W., Zhang, N., Zhao, Y., et al. (2019). A cerebellopontine angle mouse model for the investigation of tumor biology, hearing, and neurological function in NF2-related vestibular schwannoma. Nat. Protoc. *14*, 541–555. https://doi.org/10.1038/s41596-018-0105-7.

69. Daskalou, D., Romano, E., Neveü, S., Tsoutsou, P., Koutsouvelis, N., Rousset, F., Guinand, N., Becker, M., Senn, P., and Tran, S. (2026). Vestibular dose predicts toxicity in stereotactic radiosurgery for vestibular schwannomas. Clin. Transl. Radiat. Oncol. *57*, 101105. https://doi.org/10.1016/j.ctro.2025.101105.

70. Flaherty, J.P., Fairfield, H.E., Spruce, C.A., McCarty, C.M., and Bergstrom, D.E. (2011). Molecular characterization of an allelic series of mutations in the mouse Nox3 gene. Mamm. Genome *22*, 156–169. https://doi.org/10.1007/s00335-010-9309-z.

71. Fuller, P.M., Jones, T.A., Jones, S.M., and Fuller, C.A. (2004). Evidence for macular gravity receptor modulation of hypothalamic, limbic and autonomic nuclei. Neuroscience *129*, 461–471. https://doi.org/10.1016/j.neuroscience.2004.05.059.

72. Suzuki, J., Hashimoto, K., Xiao, R., Vandenberghe, L.H., and Liberman, M.C. (2017). Cochlear gene therapy with ancestral AAV in adult mice: complete transduction of inner hair cells without cochlear dysfunction. Sci. Rep. *7*, 45524. https://doi.org/10.1038/srep45524.

73. Dorr, A.E., Lerch, J.P., Spring, S., Kabani, N., and Henkelman, R.M. (2008). High resolution three-dimensional brain atlas using an average magnetic resonance image of 40 adult C57Bl/6J mice. Neuroimage *42*, 60–69. https://doi.org/10.1016/j.neuroimage.2008.03.037.

74. Presotto, L., Bettinardi, V., Mercatelli, D., Picchio, M., Morari, M., Moresco, R.M., and Belloli, S. (2022). Development of a new toolbox for mouse PET–CT brain image analysis fully based on CT images and validation in a PD mouse model. Sci. Rep. *12*, 15822. https://doi.org/10.1038/s41598-022-19872-4.

75. Montgomery, S.C., and Cox, B.C. (2016). Whole Mount Dissection and Immunofluorescence of the Adult Mouse Cochlea. J. Vis. Exp. 53561. https://doi.org/10.3791/53561.

76. Fang, Q.J., Wu, F., Chai, R., and Sha, S.H. (2019). Cochlear Surface Preparation in the Adult Mouse. J. Vis. Exp. 60299. https://doi.org/10.3791/60299.

77. Eaton-Peabody Laboratories Histology Core Mass Eye and Ear. Mapping Cochlear Length to Cochlear Frequency. https://masseyeandear.org/research/otolaryngology/eaton-peabody-laboratories/histology-core.

78. Keithley, E.M., Canto, C., Zheng, Q.Y., Wang, X., Fischel-Ghodsian, N., and Johnson, K.R. (2005). Cu/Zn superoxide dismutase and age-related hearing loss. Hear. Res. *209*, 76–85. https://doi.org/10.1016/j.heares.2005.06.009.

79. Kishimoto, I., Okano, T., Nishimura, K., Motohashi, T., and Omori, K. (2019). Early Development of Resident Macrophages in the Mouse Cochlea Depends on Yolk Sac Hematopoiesis. Front. Neurol. *10*, 1115. https://doi.org/10.3389/fneur.2019.01115.

OMTN, Volume *37*

Supplemental information

NADPH oxidase 3 inhibition preserves hearing

in mice after stereotactic radiosurgery

Dimitrios Daskalou, Francis Rousset, Stéphanie Sgroi, Lucie Oberhauser, Jean-Philippe Thiran, Constantin Tuleasca, Ileana O. Jelescu, Marc Levivier, and Pascal Senn

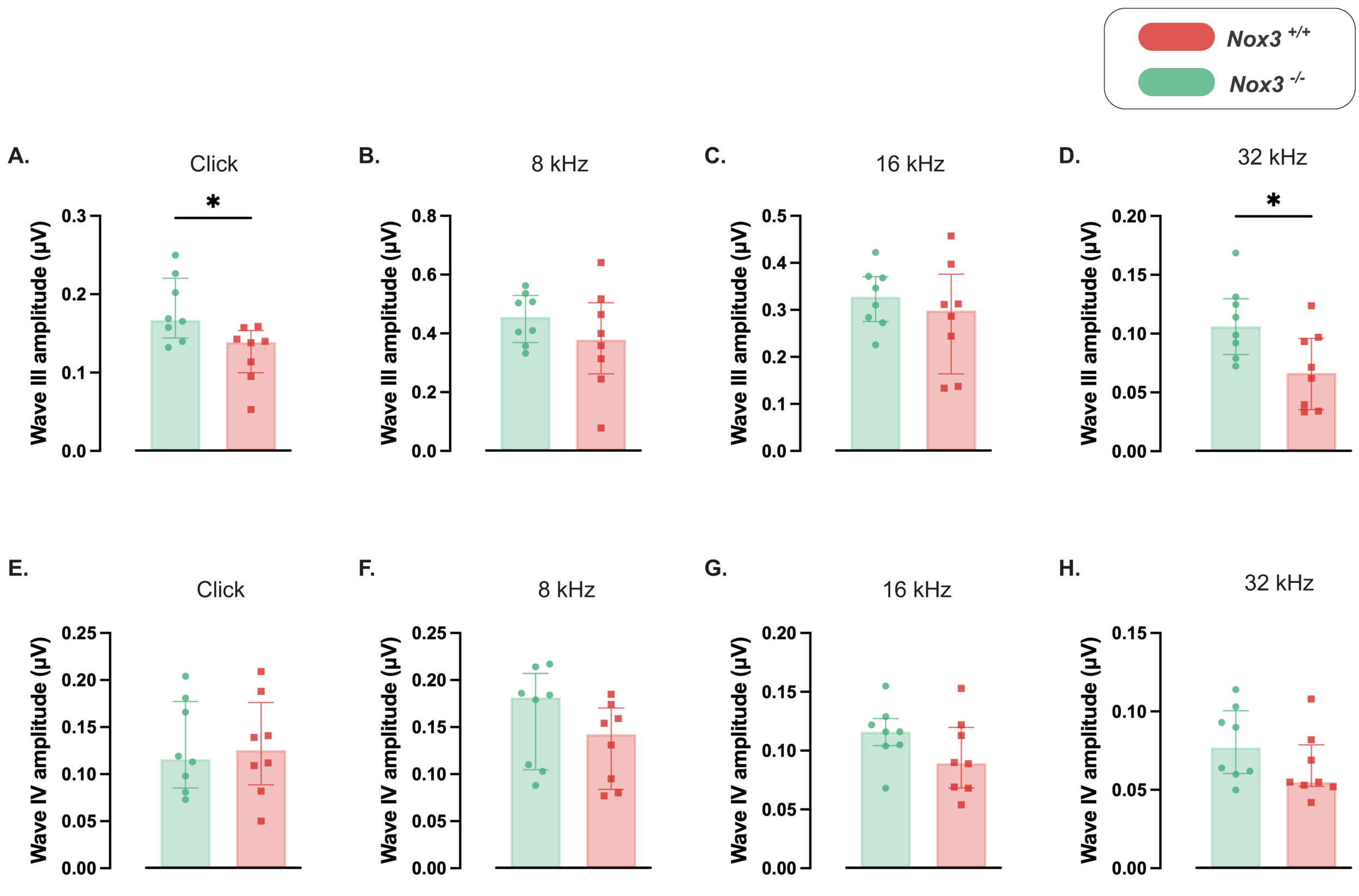

Figure S1 Auditory brainstem response wave III and IV amplitudes at week 4 post-irradiation in wild-type and Nox3-deficient mice.

(A–D) Auditory brainstem response (ABR) wave III amplitudes from the irradiated ears of *Nox3*$^{+/+}$ and Nox3$^{-/-}$ mice (n = 8 per group), measured in response to click (A) and tone bursts at 8 kHz (B), 16 kHz (C), and 32 kHz (D). Median wave III amplitudes were significantly lower in *Nox3*$^{+/+}$ mice after click and 32 kHz stimulation.

(E–H) ABR wave IV amplitudes under the same stimulation conditions. No significant differences were observed between the two groups.

Data are presented as medians with interquartile ranges. Asterisks indicate statistical significance (*$p < 0.05$). Statistical comparisons were performed using Mann–Whitney tests.

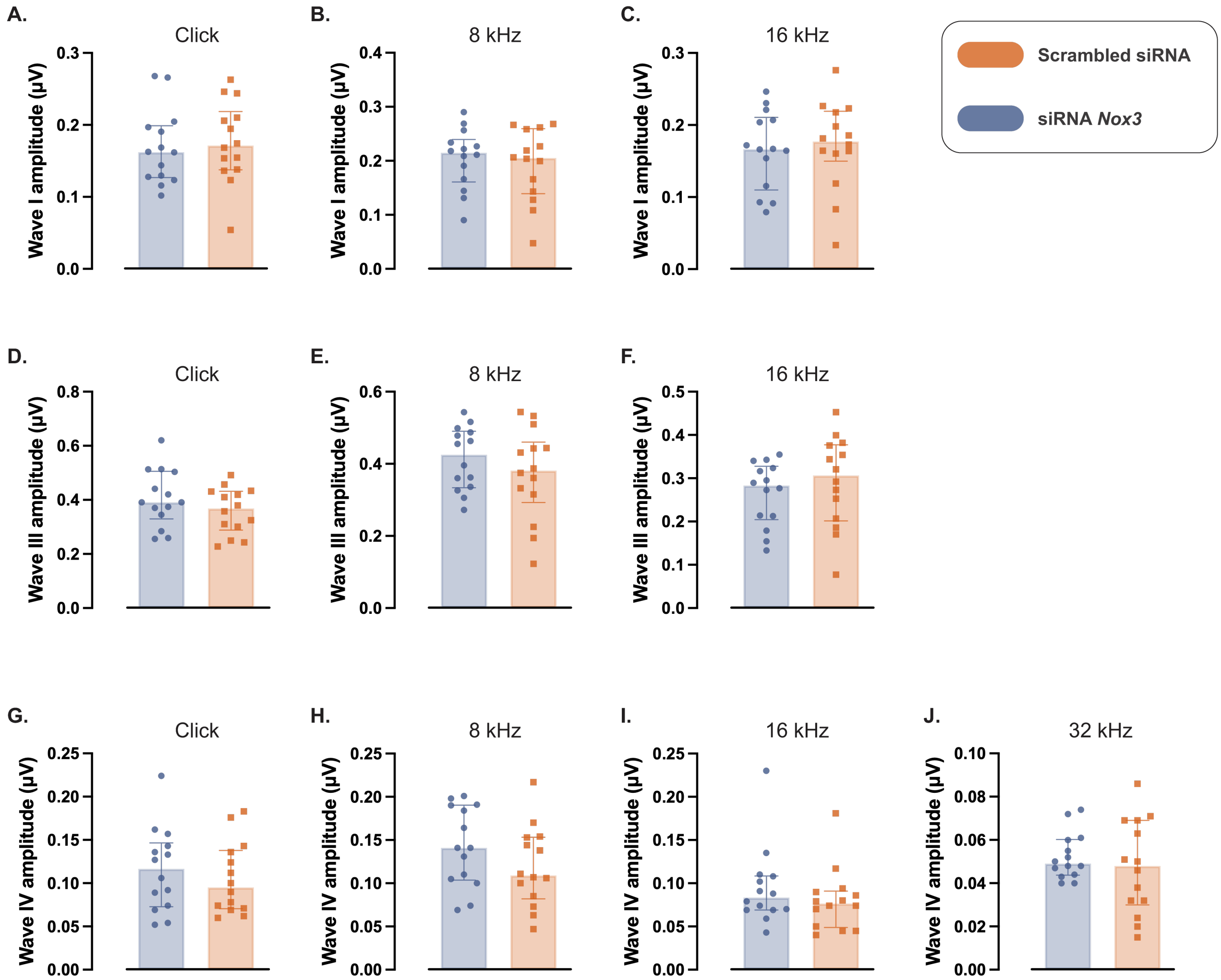

Figure S2 Auditory brainstem response wave I, III, and IV amplitudes at week 4 post-irradiation in siRNA-treated mice.

(A–C) Auditory brainstem response (ABR) wave I amplitudes from the irradiated ears of siScr- and siNox3-treated mice (n = 14 per group), measured in response to click (A) and tone bursts at 8 kHz (B) and 16 kHz (C).

(D–F) ABR wave III amplitudes under the same stimulation conditions.

(G–J) ABR wave IV amplitudes in response to click (G) and tone bursts at 8 kHz (H), 16 kHz (I), and 32 kHz (J).

No significant differences in wave amplitudes were observed between siScr- and siNox3-treated groups except for wave I and III at 32 kHz, which are shown in the main figure (Figures 2F and 2G).

Data are presented as medians with interquartile ranges. Statistical comparisons were performed using Mann–Whitney tests.

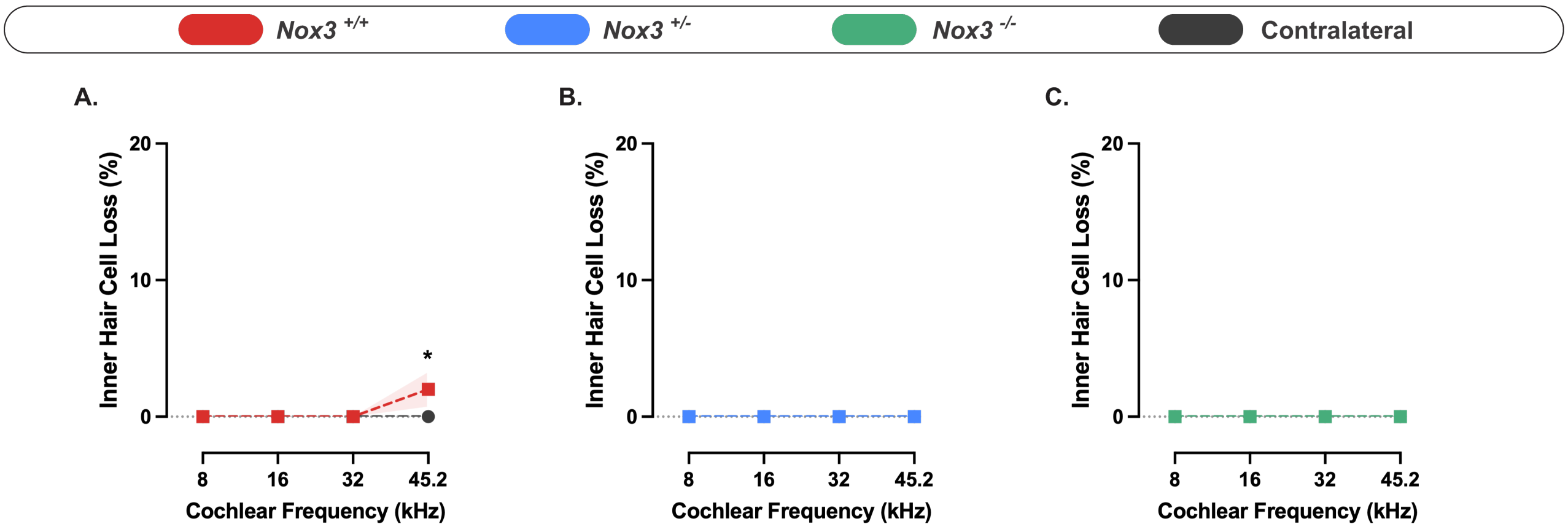

Figure S3 Inner hair cell loss across cochlear frequencies at 4 weeks post-irradiation in Nox3 genotype groups.

(A–C) Percentage inner hair cell (IHC) loss in wild-type (*Nox3*$^{+/+}$; A), heterozygous (*Nox3*$^{+/-}$; B), and *Nox3*-deficient (*Nox3*$^{-/-}$; C) mice (n = 4 per group), relative to the contralateral cochlea. IHCs were preserved in all groups except for a minor loss at 45.2 kHz in *Nox3*$^{+/+}$ mice.

Data are presented as means with shaded areas representing SEM. Asterisks indicate statistical significance (*$p < 0.05$). Statistical comparisons were performed using two-way repeated measures ANOVA with Bonferroni correction for post hoc pairwise comparisons.

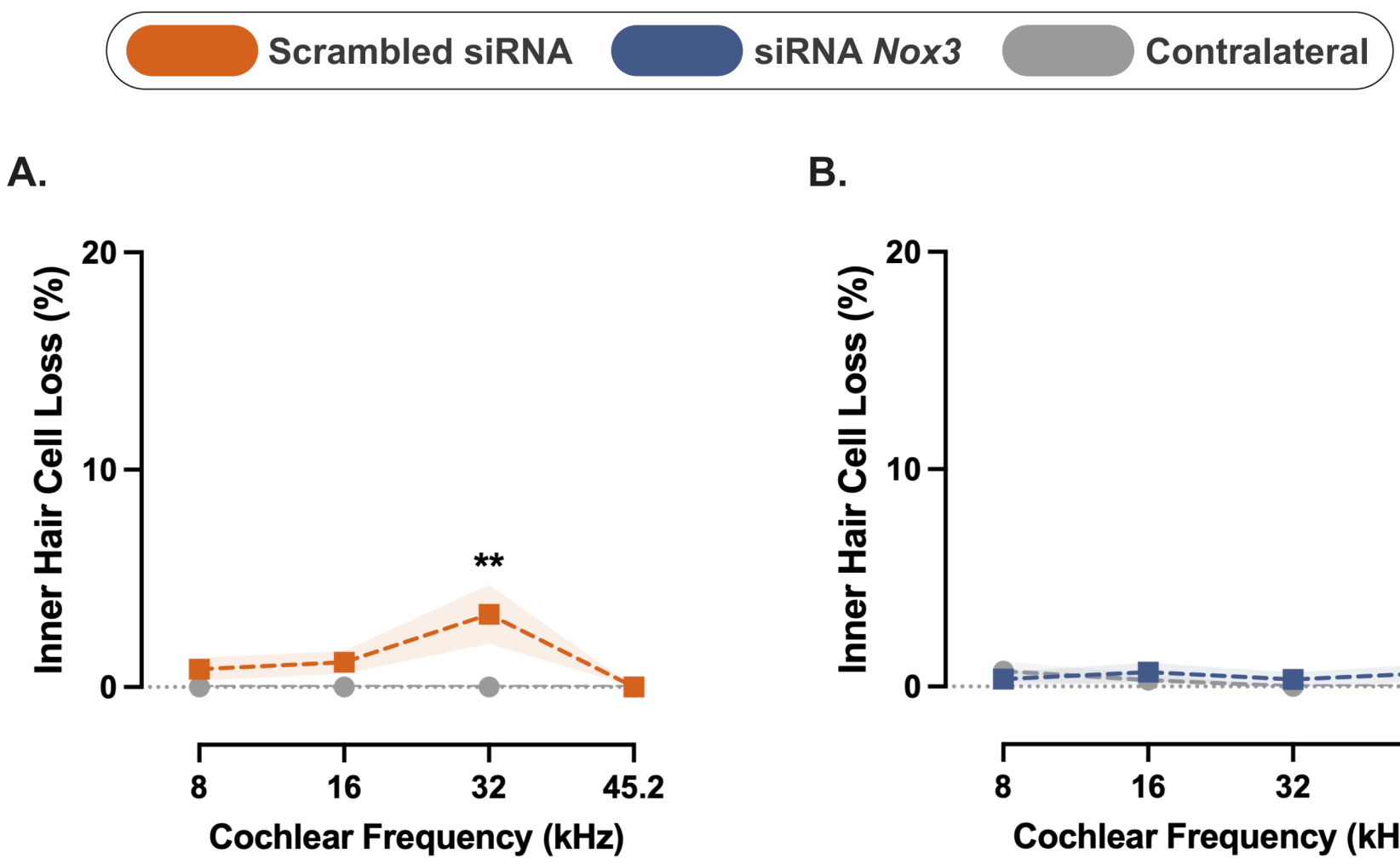

Figure S4 Inner hair cell loss across cochlear frequencies at 4 weeks post-irradiation in siRNA-treated mice.

(A–B) Percentage inner hair cell (IHC) loss in scrambled siRNA (siScr; A; n = 7) and siRNA targeting *Nox3* (siNox3; B; n = 8) groups, relative to the contralateral cochlea. IHCs were preserved in both groups, except for a minor loss at 32 kHz in siScr-treated mice.

Data are presented as means with shaded areas representing SEM. Asterisks indicate statistical significance (**$p < 0.01$). Statistical comparisons were performed using two-way repeated measures ANOVA with Bonferroni correction for post hoc pairwise comparisons.

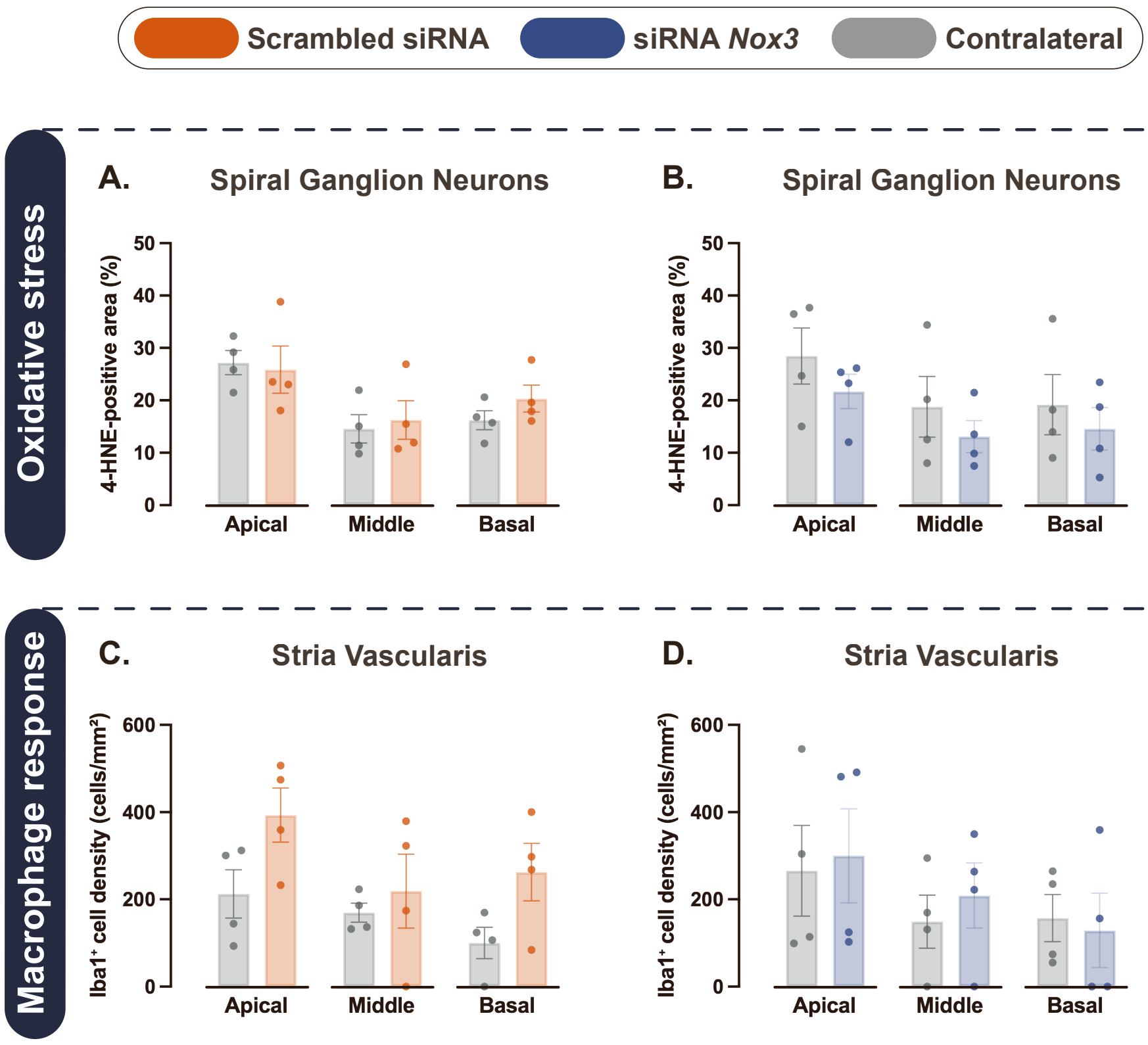

Figure S5 Oxidative stress and macrophage response in spiral ganglion neurons and stria vascularis at 4 weeks post-irradiation in siRNA-treated mice.

(A, B) Percentage of 4-hydroxynonenal (4-HNE)-positive area in spiral ganglion neurons in scrambled siRNA (siScr; A; n = 4) and siRNA targeting Nox3 (siNox3; B; n = 4) groups, relative to the contralateral cochlea. No significant increase in 4-HNE immunoreactivity was observed in either group.

(C, D) Iba1-positive cell density (cells/mm^2) in the stria vascularis in siScr-treated (C; n = 4) and siNox3-treated (D; n = 4) groups, relative to the contralateral cochlea. Macrophage infiltration tended to be higher in siScr-treated mice, although this did not reach statistical significance, whereas no significant increase was detected in siNox3-treated animals.

Data are presented as means ± SEM. Statistical comparisons were performed using two-way repeated measures ANOVA with Bonferroni correction for post hoc pairwise comparisons.

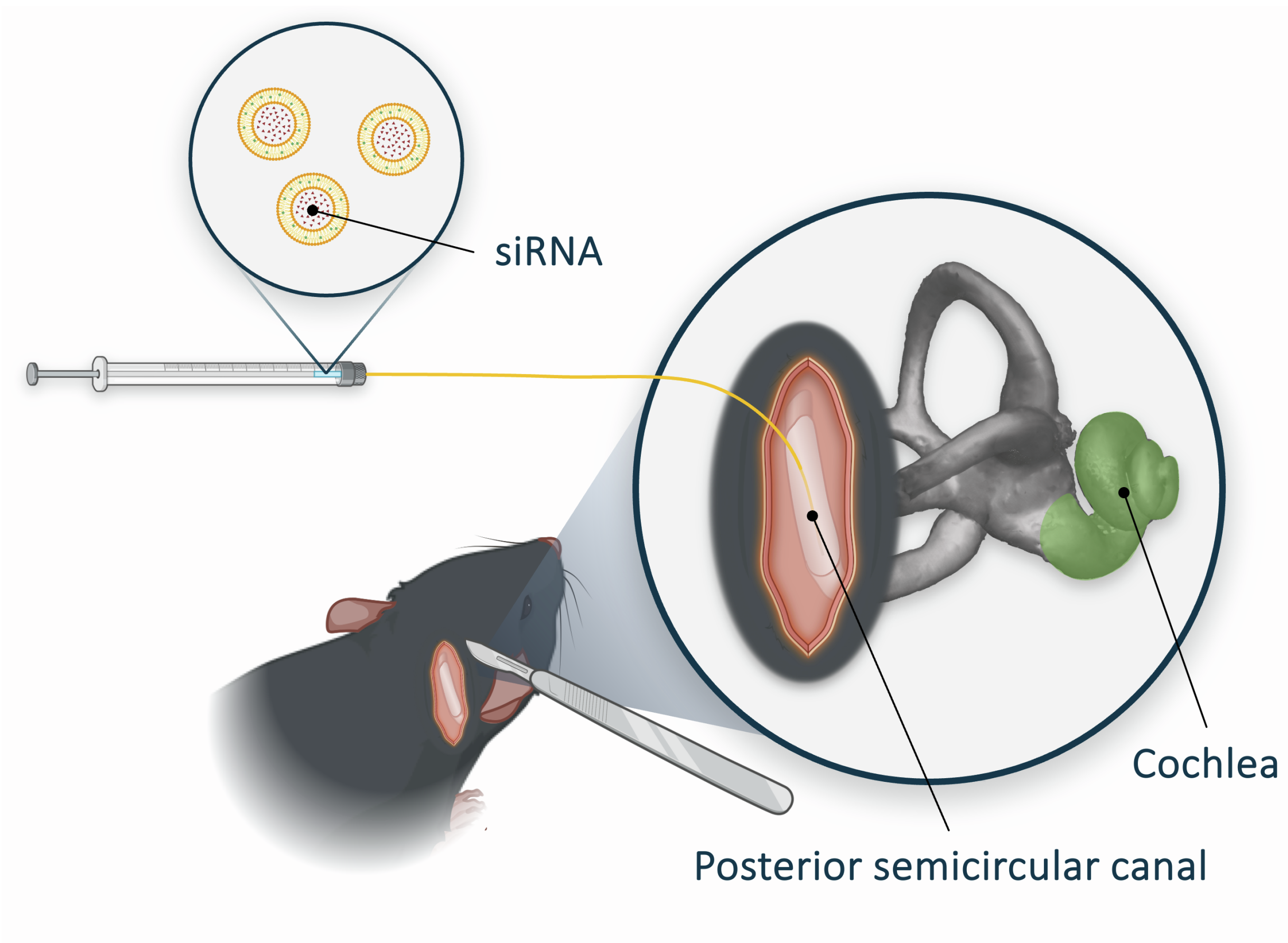

Figure S6 Schematic of siRNA delivery into the inner ear via the posterior semicircular canal. siRNA targeting *Nox3* or scrambled control siRNA was delivered into the inner ear 48 hours prior to stereotactic radiosurgery. Mice were anesthetized and placed in lateral position. Under surgical microscopy, a retroauricular incision was made, exposing the dorsal portion of the cervical trapezius muscle, located above the great auricular nerve. Following soft tissue dissection, the posterior semicircular canal was identified on the skull surface, carefully fenestrated using a 25G needle, and then cannulated. A total of 1 µL of siRNA solution was manually injected over 2 minutes through a microtube, and the injection site was sealed to prevent leakage. The wound was then closed and disinfected, and mice received local analgesia. Graphic elements adapted from BioRender.com.
